# GDP-Loaded K‑Ras Transiently Binds to Effector B‑Raf RBD, Mirroring the Structure of the Active GTP-Loaded Complex

**DOI:** 10.1021/jacs.6c09870

**Published:** 2026-08-14

**Authors:** Xinyao Xiang, Mamata Basnet, Chunhua Yuan, Lei Bruschweiler-Li, Rafael Brüschweiler

**Affiliations:** † Department of Chemistry and Biochemistry, 2647 The Ohio State University, Columbus, Ohio 43210, United States; ‡ Campus Chemical Instrument Center, The Ohio State University, Columbus, Ohio 43210, United States; § Department of Biological Chemistry and Pharmacology, The Ohio State University, Columbus, Ohio 43210, United States

## Abstract

The Ras-Raf protein association initiates the downstream mitogen-activated protein kinase (MAPK) signaling cascade. This interaction depends on the Ras-nucleotide ligand, whereby Raf preferentially binds to the active guanosine triphosphate (GTP)-loaded state over the guanosine diphosphate (GDP)-loaded state of Ras. Whether GDP-bound Ras can also form specific protein–protein interactions with Raf, however, remains unclear. Here, we characterize the interaction between human K-Ras in both nucleotide states and the Ras-binding domain (RBD) of B-Raf by solution NMR. Active K-Ras·GTP forms a tight, conformationally restricted complex with RBD, causing pronounced chemical shift perturbations at the binding interface consistent with existing cryo-EM and X-ray crystallography structures. Surprisingly, we detect specific binding between “inactive” wild-type K-Ras·GDP with RBD in the millimolar affinity range, whereby the oncogenic G12D mutant of K-Ras further strengthens this interaction. NMR relaxation dispersion and chemical exchange saturation transfer (CEST) experiments allow the detailed structural characterization of the transient K-Ras·GDP·RBD complex, along with the determination of the interaction affinity and kinetics. The results demonstrate that the transient K-Ras·GDP·RBD bound state closely resembles the active K-Ras·GTP·RBD complex, providing a quantitative understanding at the backbone ^15^N-level of the nucleotide-specific K-Ras-Raf interaction. These findings underscore the prospect of the highly adaptable GDP-bound state of K-Ras for both signaling and as a drug target.

## Graphical abstract

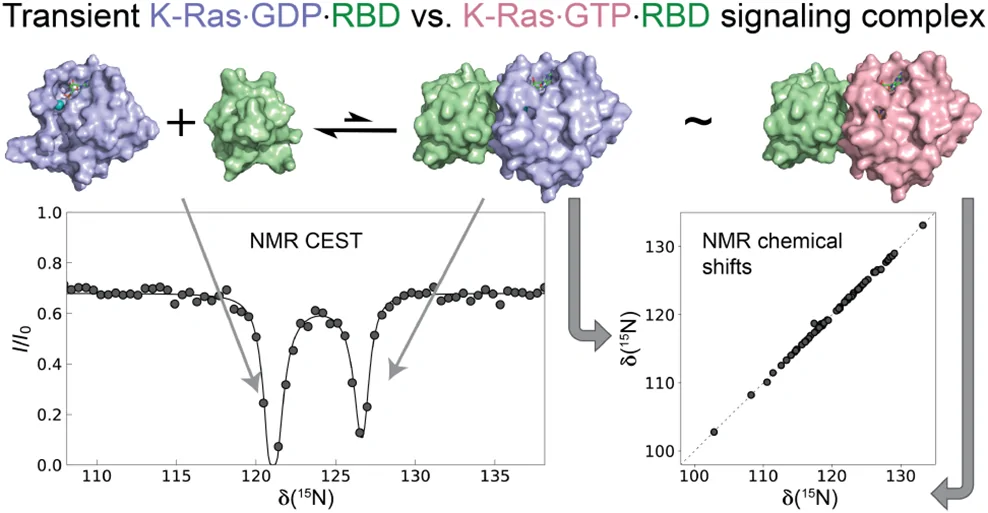

## Introduction

The small GTPase enzyme K-Ras is a key molecular switch that regulates multiple cellular signaling pathways controlling cell proliferation and differentiation.^1–3^ Like other Ras-family proteins, K-Ras functions by cycling between an active, GTP-bound “on” state and GDP-bound “off” state believed to be signaling inactive (Figure 1). This cycle is regulated by helper proteins, namely guanine nucleotide exchange factors (GEFs) and GTPase-activating proteins (GAPs).^2,4,5^ While K-Ras is capable of hydrolyzing GTP and thereby deactivating itself, its intrinsic hydrolysis rate is relatively slow, and it relies on GAP to accelerate this process. GEFs, on the other hand, promote the dissociation of GDP to enable GTP loading and activate K-Ras. Oncogenic mutations disrupt this cycle by locking K-Ras in the active state, resulting in continuous downstream signaling leading to uncontrolled cell growth. In fact, K-Ras is the predominant Ras isoform mutated in cancer, with nearly 75% of all Ras mutations occurring in K-Ras, while Ras mutations overall are found in approximately 19% of human cancers.^6^

**Figure 1:**
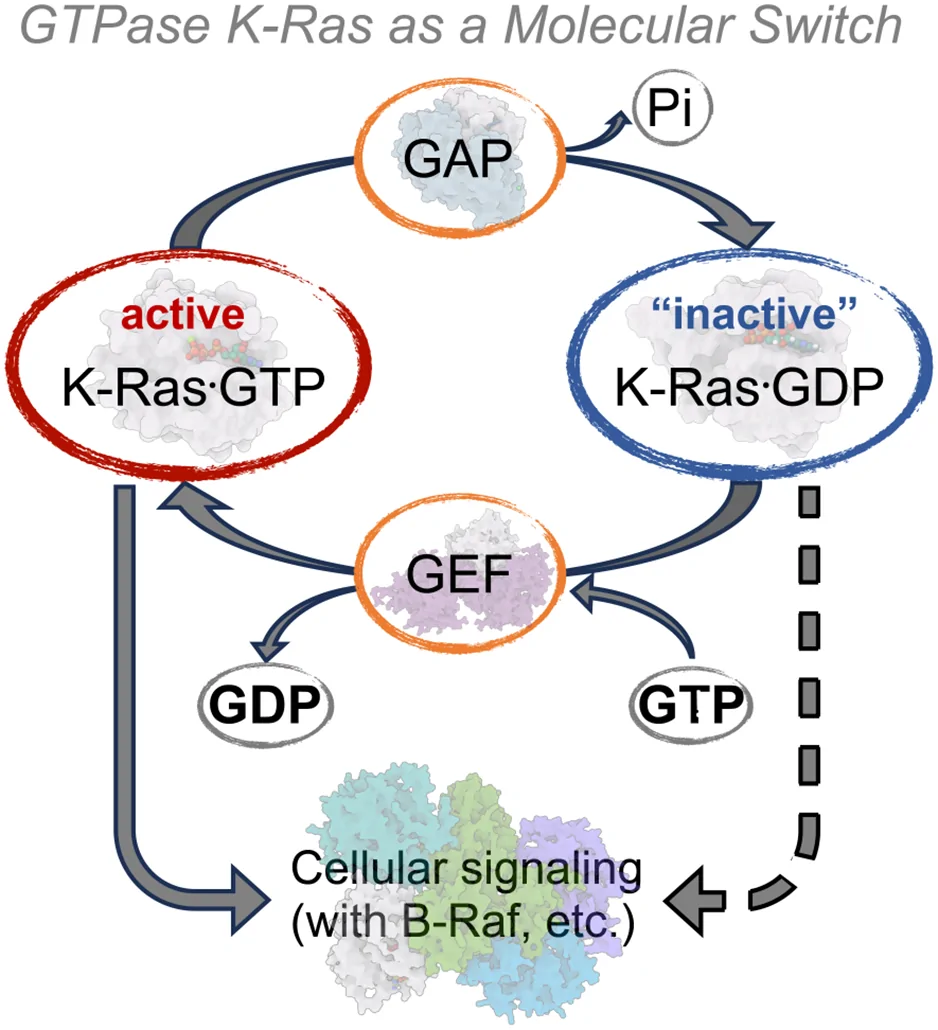
K-Ras acts as a molecular switch by cycling through the active GTP-bound state and inactive GDP-bound state. K-Ras·GTP binds downstream effector proteins, such as B-Raf, and triggers cell proliferation and differentiation. Surprisingly, K-Ras·GDP can also weakly yet specifically interact with B-Raf, as demonstrated below. The K-Ras and complex structures are from PDB ID 5W22 (K-Ras·GTP), 4OBE (K-Ras·GDP), 6OB2 (K-Ras·GNP·GAP-related domain of neurofibromin), 6EPL (K-Ras·SOS1), and 8DGS (K-Ras·GNP·B-Raf·MEK1·14–3–3) and the illustrations are generated using Mol*.^83,84^

The structural dynamics of K-Ras in its GTP- and GDP-bound states differ primarily within the effector lobe (residues 1–86), particularly in the Switch I (residues 30–38) and Switch II (residues 60–76) regions.^7,8^ These regions are functionally critical because they form the interaction interface with downstream effectors as well as regulatory proteins, as shown in previous studies.^9–17^ Thus, differences in the structural dynamics of the Switch regions provide the basis for the nucleotide-dependent selectivity of K-Ras interactions with its binding partners. We recently reported the near complete backbone resonance assignments of K-Ras·GTP and K-Ras·GDP and, for the first time, directly detected the Switch regions in the native GTP-bound K-Ras,^8,18^ which had previously been unobservable by both crystallography and NMR.^19,20^ These advances enabled a comprehensive characterization of K-Ras dynamics using Carr–Purcell–Meiboom–Gill (CPMG) relaxation dispersion, chemical exchange saturation transfer (CEST), and nanoparticle-assisted spin relaxation (NASR) experiments. GTP-bound Ras has long been known to undergo millisecond time scale conformational exchange between two states, termed state 1 and state 2.^21–23^ In K-Ras·GTP, the minor excited state (state 1), populated at around 10% for wild-type (WT), resembles the GDP-bound state with flexible and partially disordered Switch regions, whereas the ground state (state 2) is relatively rigid and competent for downstream signaling.^8^

The Raf serine/threonine kinase was the first downstream effector of Ras identified and provides a direct link between Ras activation and the MAPK signaling cascade.^24,25^ The Raf family comprises three isoforms, A-Raf, B-Raf, and C-Raf (Raf1), and they share three conserved regions, CR1, CR2, and CR3.^26^ The N-terminal CR1 region contains the Ras-binding domain (RBD) and the cysteine-rich domain (CRD), which together mediate Ras recognition and membrane association.^14,16,27^ Raf preferentially recognizes and interacts with the GTP-bound form of Ras via RBD, an interaction involved in the recruitment of Raf to the plasma membrane. At the membrane, Raf undergoes intramolecular rearrangements that relieve autoinhibition and promote activation of the kinase domain.^28,29^ The role of the CRD is now understood to extend beyond membrane association, as the crystal structure of K-Ras bound to GTP analog GNP in complex with C-Raf RBD-CRD showed that the CRD can contact K-Ras directly and expand the Ras-Raf interaction interface.^14^ A more recent cryo-EM study captured K-Ras·GNP bound to autoinhibited B-Raf·MEK1·14–3–3 assemblies, providing structural details of a preactivation recruitment intermediate.^16^ In addition, interactions between Ras·GNP and A-Raf as well as C-Raf RBD have been monitored directly in solution by NMR.^30,31^ Despite extensive studies on Ras·GTP-Raf interactions, the interaction propensity between GDP-bound Ras and Raf has remained unclear. Most recently, Ikura and coworkers reported that B-Raf RBD exhibits low-affinity binding with the G12D mutant of K-Ras·GDP.^32^ However, the microscopic nature of this unexpected K-Ras-effector interaction and whether it is nonspecific or specific involving a structurally well-defined complex remains unknown.

Here, we investigate the interaction between B-Raf RBD and K-Ras in both of its native GTP and GDP nucleotide-bound states by solution NMR spectroscopy. We report near complete backbone resonance assignment of the stable WT K-Ras·GTP·RBD complex and map the interaction interface through analyzing chemical shift perturbations. In contrast, K-Ras·GDP interacts transiently with RBD, which is accompanied by peak broadening. By applying ^15^N CPMG and CEST experiments to probe this weak association, affinity and kinetics information on the interaction is obtained together with chemical shifts of the transiently formed complex, thereby providing also structural insight into complex formation. The results allow direct comparison between the structure of the K-Ras·GDP·RBD bound state with the active GTP-bound complex and how GTP vs GDP nucleotide and WT vs oncogenic mutant G12D modulate the Ras-effector interaction.

## Experimental Section

### Protein Expression and Sample Preparation

WT and G12D human K-Ras4B G-domain (residues 1–169) were expressed and purified as described previously.^8^ The gene encoding the Ras-binding domain (RBD) of human B-Raf (residues 155–227) was synthesized by IDT Inc., and amplified by PCR from the corresponding DNA template and subcloned into the bacterial expression vector pTBSG1.^33^ The resulting construct was verified by Sanger sequencing before being transformed into chemically competent *E. coli* BL21­(DE3) cells for recombinant protein expression. The overexpression was carried out in M9 minimal media with ^15^NH_4_Cl (1 g L^–1^) as the sole nitrogen source for uniformly ^15^N-labeled proteins, and ^13^C glucose (4 g L^–1^) and ^15^NH_4_Cl (1 g L^–1^) as the sole carbon and nitrogen sources for uniformly ^13^C,^15^N-labeled proteins. Protein expression was induced with 0.5 mM IPTG at OD_600_ of 1.0–1.2 and incubated at 25 °C overnight. The proteins were purified with Ni-NTA affinity resins as described previously.^33^

The purified RBD was buffer exchanged to 20 mM HEPES buffer (pH 7.0) with 100 mM NaCl and supplemented with 2 mM reducing agent tris­(2-carboxyethyl)­phosphine (TCEP). K-Ras·GDP and K-Ras·GTP samples were prepared as described previously.^8^ For K-Ras·GTP·RBD complex samples, K-Ras·GTP was mixed with equimolar RBD, and the final concentration of the complex was around 0.7 mM. The final samples were in 20 mM HEPES (pH 7.0) buffer with 100 mM NaCl, 10 mM GTP (Fisher Scientific), 5 mM MgCl_2_, 2 mM TCEP. For K-Ras·GDP and RBD mixtures, K-Ras·GDP was mixed with RBD with final concentrations listed in Table 1. The final samples were in 20 mM HEPES buffer (pH 7.0) with 100 mM NaCl, 5 mM GDP (Sigma), 5 mM MgCl_2_, 2 mM TCEP. All samples contained 5% D_2_O (v/v) for NMR field lock.

**Table 1:** Summary of Sample Concentrations and Binding Parameters Derived from Amide ^15^N CEST and CPMG Data

Protein^a^ (^15^N-labeled)	Ligand^a^ (unlabeled)	*K*_D_ ^b^ (mM)	Residence time^c^ (τ_res_ = 1/*k*_off_) (ms)	*k*_ex_ ^c^ (s^–1^)	*k*_on,app_ (s^–1^)	*k*_on_ (mM^–1^ s^–1^)	*k*_off_ (s^–1^)	*p*_b_ ^c^ (%)
0.60 mM	0.15 mM	1.56 ± 0.24	6.39 ± 0.23	167.7 ± 5.9	11.1	100.6	156.6	6.62 ± 0.12
WT K-Ras·GDP	RBD							
0.33 mM	0.09 mM	0.38 ± 0.10	14.7 ± 1.5	77.1 ± 7.3	9.1	177.3	68.0	11.8 ± 1.1
G12D K-Ras·GDP	RBD							
0.55 mM	0.55 mM	3.86 ± 0.89	6.82 ± 0.13	165.4 ± 2.8	18.7	38.0	146.7	11.30 ± 0.27
RBD	WT K-Ras·GDP							
0.51 mM	0.25 mM	1.83 ± 0.47	12.41 ± 0.45	89.4 ± 3.1	8.8	44.0	80.6	9.84 ± 0.34
RBD	G12D K-Ras·GDP							

a Concentrations were determined by UV absorption at 280 nm except for the 0.33 mM G12D K-Ras·GDP and 0.09 mM RBD sample, which were determined by ^1^H PULCON experiments (d1 = 30 s and 1 mM DSS as reference).

b Errors represent 1 s.d. and are derived from the analytical error propagation of the uncertainties in *p*_b_ and concentrations, where the errors in *p*_b_ are from 500-time bootstrap simulations in CEST and CPMG global fits with concentration uncertainties assumed to be 10% for RBD and 20% for K-Ras.

c Errors represent 1 s.d. and are from 500-time bootstrap simulations in CEST and CPMG global fits.

### Resonance Assignments

All NMR experiments, including the resonance assignment experiments and the dynamics experiments described below, were performed at 298 K on a Bruker magnet with an AVANCE III console operating at 850 MHz (19.97 T) equipped with a 5 mm TCI cryoprobe. For the sequence specific backbone resonance assignments, standard triple-resonance experiments (3D HNCO, HNCOCA, and HNCA)^34^ were collected on the following three sets of samples: uniformly ^15^N,^13^C-labeled WT K-Ras·GTP in complex with equimolar unlabeled RBD, uniformly ^15^N,^13^C-labeled RBD with equimolar unlabeled G12D K-Ras·GTP, and ^15^N,^13^C-labeled RBD alone. All triple-resonance experiments were recorded using sensitivity-enhanced gradient coherence selection,^35,36^ semiconstant time acquisition in the ^15^N dimension,^37^ and Poisson gap nonuniform sampling.^38^ In addition, 3D CNH-NOESY experiments^39^ with mixing times set to 180 ms were collected to assist the resonance assignments. Experiments were started with freshly purified samples, and samples containing enzymatically active K-Ras·GTP were exchanged by fresh samples once the hydrolyzed peaks became pronounced. All the data were processed using NMRPipe^40^ with SMILE^41^ for NUS reconstruction, and visualized using NMRViewJ,^42^ both via NMRBox.^43^ The backbone assignment of free RBD was transferred from BMRB^44^ entries 17030 and 30047 and confirmed with the above-mentioned experiments.

### Relaxation Dispersion and CEST Experiments

Backbone amide ^15^N-(ST-CW-)­CPMG NMR relaxation dispersion^45^ and ^15^N-CEST experiments^46^ were performed for ^15^N-labeled K-Ras·GDP in the presence of unlabeled RBD and ^15^N-labeled RBD in the presence of unlabeled K-Ras·GDP, for both WT K-Ras and G12D mutant. The protein concentrations used are summarized in Table 1. Amide HSQC/HMQC experiments^47^ were measured to assist the sign determination of the chemical shift differences Δϖ during the exchange fittings. In addition, ^15^N CPMG and ^15^N CEST experiments were recorded on a ^15^N-labeled RBD sample, and ^15^N CPMG was recorded on a freshly made WT K-Ras·GTP·RBD complex sample. All dynamics experiments were measured with recovery delay d1 of 2 s. The constant relaxation time *T*_CPMG_ for the CPMG block was set to 30 or 40 ms. The CPMG ^15^N-refocusing pulses were applied with a B_1_-field of approximately 6.1 kHz, and the CPMG pulsing frequency ν_CPMG_ was varied from 25 Hz to 2 kHz. For CEST experiments, mixing times ranged from 150 to 250 ms with the saturation field strength B_1_ set around 20 Hz. The calibrated^48^ B_1_-field strength, CEST saturation time, and *T*_CPMG_ used for each sample are listed in Table S2.

The dynamics NMR experiments were processed with NMRPipe^40^ and visualized with NMRFAM-SPARKY or POKY.^49,50^ The cross-peak intensities were extracted with either autoFit in NMRPipe or Voigt fitter.^51^ CPMG and CEST profiles were analyzed collectively using ChemEx.^46^ For the four K-Ras·GDP and RBD mixture samples, the profiles were fitted to a two-state exchange model using the two-step procedure described previously.^8^ Briefly, in the first step, residue-specific ^15^N *R*_ex_ values were estimated from the CPMG profiles as the difference between the effective *R*_2_ values at the minimal and maximal ν_CPMG_ frequencies (*R*_ex_ = *R*_2,eff_(min­(ν_CPMG_)) – *R*_2,eff_(max­(ν_CPMG_)). CEST and CPMG profiles of residues with *R*_ex_ > 5 s^–1^ were then selected to determine the global exchange parameters with ChemEx.^46^ ChemEx allows numerical simulation of the CEST and CPMG pulse sequences with a two-state exchange model. The output includes the global parameters exchange rate *k*_ex_ = *k*_AB_ + *k*_BA_ and populations of each state *p*_A_ and *p*_B_ = 1 – *p*_A_ (subscripts A and B stand for state A and B), as well as the site-specific chemical shift differences Δϖ. In the second step, CEST and CPMG profiles of all residues were analyzed with the global parameters *k*_ex_ and *p* determined in step 1 constrained to obtain Δϖ of each residue. The sign of Δϖ was obtained from CEST and/or HSQC/HMQC experiments. The errors in peak intensities were estimated from replicate planes in CPMG or CEST experiments, which were then propagated through the analysis. Errors in the global exchange parameters and Δϖ were determined from 500 bootstrap replicate simulations.

### Conversion of Global Exchange Parameters to Binding Parameters

The two states identified in CEST and CPMG analysis correspond to the free and bound states of the K-Ras·GDP and RBD binding process. For convenience, we refer below to the NMR detectable ^15^N-labeled protein in the sample as the “protein” and the unlabeled protein as the “ligand”. For the experimental conditions used in this work, we identified the dominant state to be the free protein and the minor state to be the protein bound to unlabeled ligand (see Results and Discussion Sections). Therefore, the population of the minor state (*p*_B_) is equal to the bound population *p*_b_. The exchange rate *k*_ex_ is *k*_ex_ = *k*_AB_ + *k*_BA_ = *k*_on,app_ + *k*_off_ where the apparent association rate is *k*_on,app_ = [L]_eq_ *k*_on_ and residence time of the bound state τ_res_ = 1/*k*_off_ = 1/[(1 – *p*_b_) *k*_ex_]. Both *p*_b_ and *k*_on,app_ depend on the protein and ligand concentrations in the sample and can be externally controlled. Using sub-mM protein concentrations, we could tune *p*_b_ to 5–15% by establishing concentration ratios [P]/[L] in a range suitable for CPMG and CEST measurements. For known initial protein and ligand concentrations, [P]_0_ and [L]_0_, and *p*_b_ determined from CEST/CPMG experiments, the dissociation constant *K*_D_ can be obtained as follows:

1 $$K_{D}=k_{off}/k_{on}=\left(1-p_{b}\right)\left({\left[L\right]}_{0}-p_{b}{\left[P\right]}_{0}\right)/p_{b}$$

The protein and ligand concentrations were determined with UV absorbance at 280 nm using the Beer–Lambert law with sequence-derived extinction coefficients, except for the ^15^N-labeled G12D K-Ras·GDP + unlabeled RBD sample for which the concentrations were determined with the ^1^H PULCON experiments.^52^ For the latter, 1 mM DSS was used as reference, and ^1^H 1D spectra were recorded with a recovery delay of 30 s. The most upfield methyl groups in the ^1^H 1D spectra of K-Ras·GDP and RBD were integrated and used for concentration determination. Error bars in *K*_D_ and 1/*k*_off_ were derived by analytically propagating the uncertainties in *p*_b_ and *k*_ex_, obtained from bootstrap simulations of the global fits, as well as the uncertainties in protein concentrations, which were assumed to be 10% for RBD and 20% for K-Ras.

## Results

### Resonance Assignments of K-Ras·GTP·RBD

The active K-Ras·GTP state triggers cellular signaling through interacting with downstream effectors, such as Raf kinases. Previous biophysical studies have shown that K-Ras bound with GTP analogs forms a stable complex with the RBD of B-Raf with a binding affinity *K*_D_ in the low-nM range.^16,53^ Consistent with this established mechanism, we observed clear binding-induced chemical shift perturbations (CSPs) in the backbone amide ^1^H–^15^N HSQC spectra for both ^15^N-labeled K-Ras with native GTP and ^15^N-labeled B-Raf RBD upon adding their corresponding equimolar unlabeled binding partners (Figure 2).

**Figure 2:**
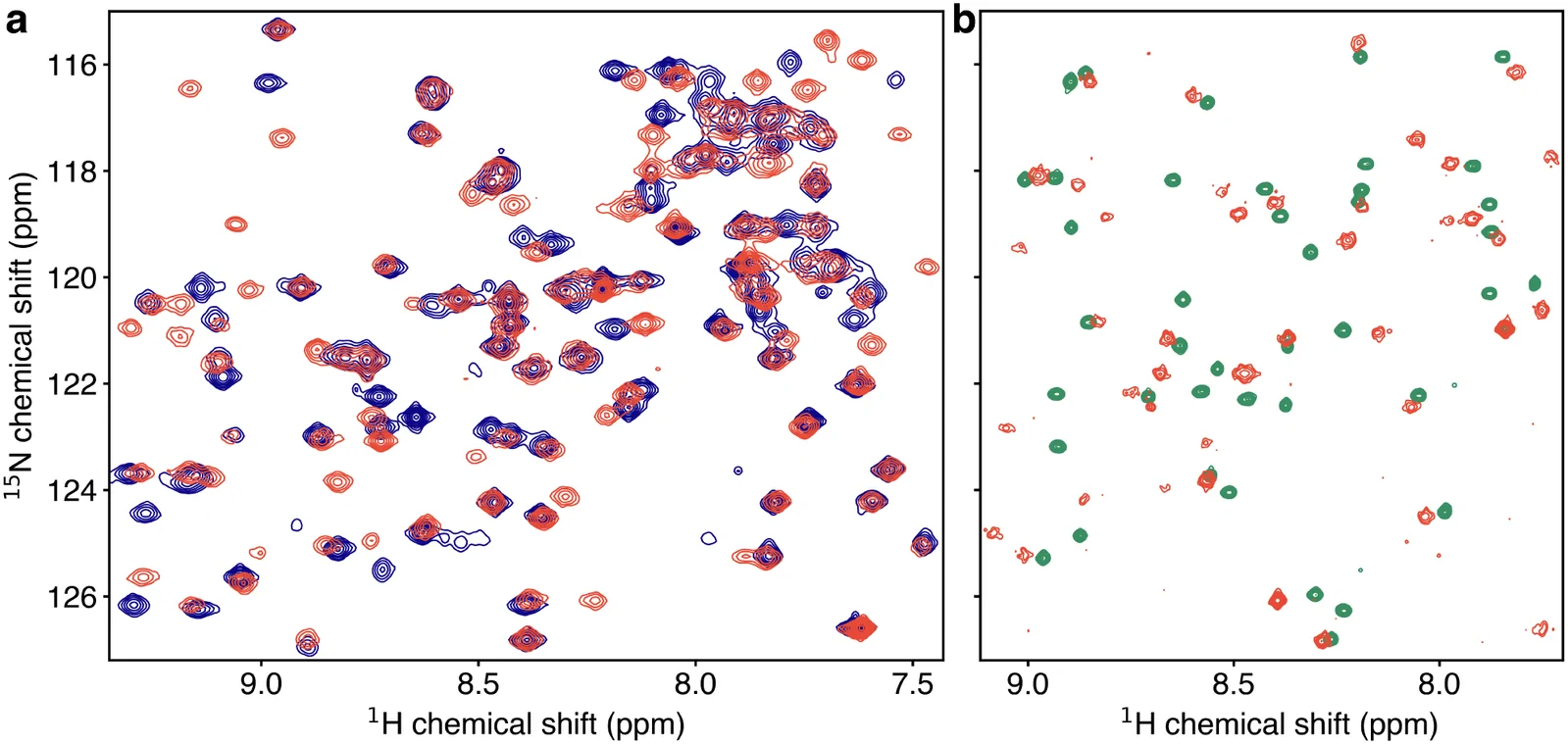
Section of amide HSQC spectra of K-Ras·GTP, B-Raf RBD, and their complex. **a**) ^1^H–^15^N HSQC of K-Ras·GTP alone (navy) and in complex with equimolar unlabeled B-Raf RBD (coral). **b**) ^1^H–^15^N HSQC of B-Raf RBD alone (green) and in complex with equimolar unlabeled K-Ras·GTP (coral). The protein concentrations were 0.7 mM for free K-Ras·GTP and for the K-Ras·GTP·RBD complex in which K-Ras·GTP was ^15^N-labeled, 0.9 mM for free RBD, and 0.4 mM for the K-Ras·GTP·RBD sample in which RBD was ^15^N-labeled. The other sample conditions for this and all other samples are listed in the Experimental Section.

The resonances of the complex are well-dispersed with peaks that are not part of the Switch regions being overall broader than those of the individual proteins due to the higher molecular weight of the complex. Nonetheless, we could obtain near complete backbone resonance assignments of the WT K-Ras·GTP·RBD complex at room temperature 298 K (98% assigned, Table S1). We then quantified the CSPs induced by the complex formation (Figure S1). Both WT and G12D K-Ras·GTP show similar changes in chemical shifts when binding with RBD with changes predominantly localized in the N-terminal effector lobe (residues 1–86). The strongest perturbation, including residues E37, D38, S39, R41, I55, and D57, occurs in the Switch I loop and adjacent β_2_ and β_3_ strands. On the effector side, B-Raf RBD, which has a ubiquitin-like 3D structure with five β-strands, one α-helix, and two 3_10_ helices,^54^ exhibits large CSPs for residues, such as for R166, V168, and M187, mostly belonging to the second β-strand and the end of the α-helix.

Next, we investigated the backbone dynamics of the complex using relaxation dispersion experiments.^55^ Unlike free K-Ras·GTP, ^15^N CPMG measurements on the ^15^N-labeled WT K-Ras·GTP sample with equimolar unlabeled RBD present showed no detectable dispersion (Figure S2). These data indicate that the complex adopts a single dominant state without any observable millisecond exchange with additional states.

### K-Ras·GDP Interactions with RBD

Although the inactive state K-Ras·GDP is generally expected to lack significant interactions with RBD, we observed minor chemical shift perturbations and substantial peak broadening for several residues in the ^1^H–^15^N HSQC spectra of ^15^N-labeled WT and G12D K-Ras·GDP following unlabeled RBD addition, even when RBD was present at a substoichiometric level relative to K-Ras. The peak broadening is most pronounced for residues located within both Switch regions, such as V29, Y32, A66, Y71, M72 (Figure 3a). To investigate the origin of this broadening, we performed backbone ^15^N-CPMG and ^15^N-CEST experiments.^45,46,55^ Figure 3 shows representative CPMG and CEST profiles of residues Y32, T35, A66, and M72, with more examples displayed in Figure S3. Since the extent of peak broadening can be modulated via *p*_b_ and *k*_ex_ by the relative concentrations of K-Ras·GDP and RBD (see Experimental Section), we optimized the protein concentrations to achieve notable broadening and relaxation dispersion, while ensuring that the broadened cross-peaks remained quantifiable. The final protein concentrations are given in Table 1.

**Figure 3:**
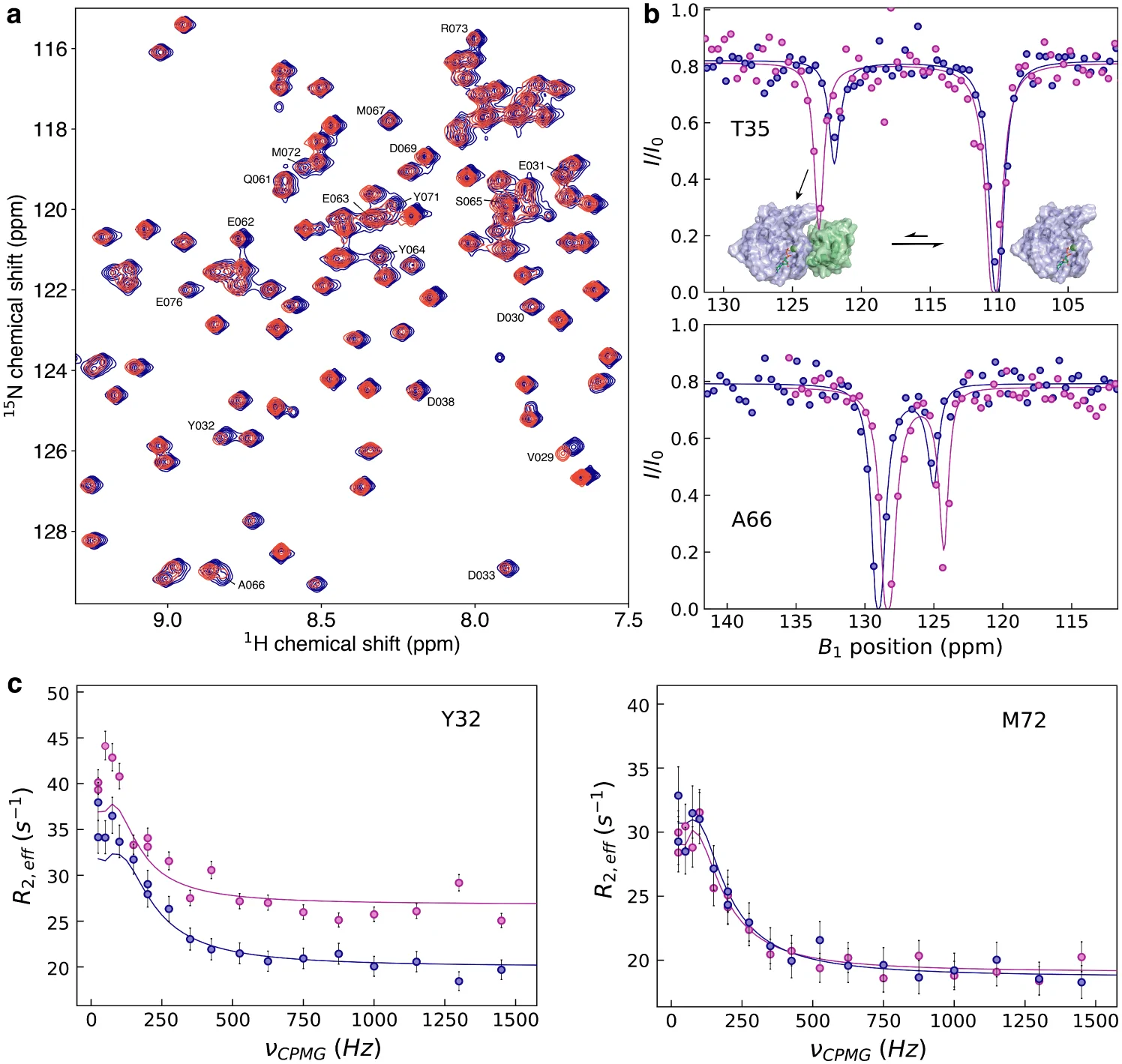
K-Ras·GDP transiently interacts with B-Raf RBD leading to the broadening of K-Ras·GDP resonances. **a**) Section of amide ^1^H–^15^N HSQC spectra of ^15^N-labeled K-Ras·GDP in the absence (navy) and in the presence of unlabeled RBD (coral) (K-Ras·GDP to RBD concentration ratio of 4:1). Subtle changes in chemical shifts along with line broadening are observed in the presence of RBD. The labeled cross-peaks belong to residues in the Switch I and II regions. **b**) ^15^N CEST profiles of selected residues in the Switch regions (T35, A66) of WT (blue) and G12D (purple) K-Ras·GDP in the presence of RBD. The major dips correspond to the free K-Ras·GDP state, which transiently binds with RBD resulting in the minor dips in the CEST profiles. The WT data set was recorded with a ^15^N CEST saturation–offset range of 95–142.4 ppm, whereas the G12D data set was recorded from 96.5 to 135.5 ppm. **c**) ^15^N CPMG profiles of selected residues in the Switch regions (Y32, M72) of WT (blue) and G12D (purple) K-Ras·GDP in the presence of RBD. Additional CEST and CPMG profiles are shown in the Supporting Information (Figure S3). Errors in *R*_2,eff_ were estimated from analytical propagation of peak intensity errors, which were derived from replicate planes in CPMG experiments.

The ^15^N CEST saturation profiles indicate the presence of two distinct protein states as evidenced by the two discrete dips observed for a series of residues, with the dominant (major) dip corresponding to the free K-Ras·GDP form. More than 40 residues show significant exchange effects in ^15^N CEST and CPMG profiles (Table S2), and their data were fitted to a two-state conformational exchange model using ChemEx software.^46^ The global fits yield the exchange rate *k*_ex_ and the population of each state, which were subsequently constrained to fit the profiles of all residues and produce residue-specific chemical shift differences Δϖ between the two exchanging protein states. All profiles can be well explained by a global two-state exchange process. Given the concentration-dependent behavior of this exchange, we could assign the low-populated conformation to K-Ras·GDP bound to RBD (K-Ras·GDP·RBD). Hence, the population of the minor state is the bound population *p*_b_ and *k*_ex_ = *k*_on,app_ + *k*_off_ of the binding process. We then determined the residence time, τ_res_ = 1/*k*_off_, of the bound state from *p*_b_ and *k*_ex_, and together with the protein concentrations, the binding affinity *K*_D_ (see eq 1). These global fit parameters are summarized in Table 1. Both WT and G12D K-Ras·GDP form a transient complex with RBD with low millimolar binding affinity with a residence time in the low millisecond range. Interestingly, the G12D mutant has a stronger affinity than WT (*K*_D_ = 0.38 mM vs 1.56 mM) and a longer residence time (τ_res_ = 14.7 ms vs 6.39 ms).

### Chemical Shifts of Transient K-Ras·GDP·RBD

Figure 4 depicts the residue-specific ^15^N chemical shift differences Δϖ between the RBD-bound and free K-Ras·GDP derived from CPMG and CEST analysis. WT and G12D K-Ras·GDP show a similar Δϖ pattern. The majority of chemical shift changes are located within the N-terminal effector lobe, whereas the C-terminal lobe shows much smaller changes. Residues with the largest |Δϖ| values are observed in or around the Switch I region (such as H27, Y32, T35, I36, E37, S39) and the Switch II region (such as A66, Y71, M72, G75), suggesting that these regions experience conformational changes and/or form the interaction interface with RBD.

**Figure 4:**
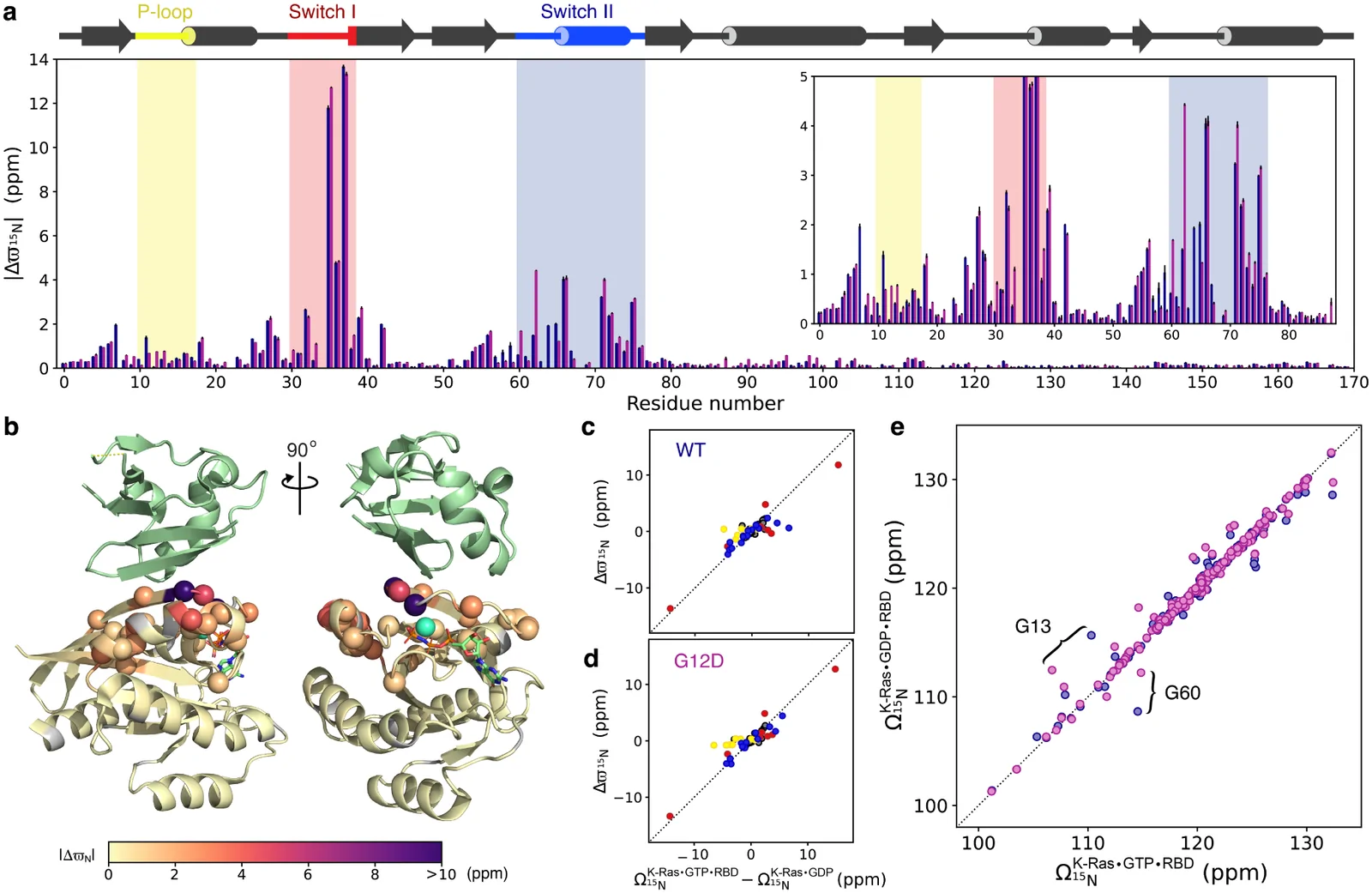
Chemical shifts of K-Ras·GDP in the RBD-bound state closely match those of RBD-bound K-Ras·GTP. **a**) Absolute ^15^N chemical shift differences |Δϖ_N_| between K-Ras·GDP in the RBD-bound state and free state, which are derived from ^15^N CEST and CPMG experiments, are shown as a function of protein sequence. WT K-Ras·GDP |Δϖ_N_| values are in dark blue and G12D in purple. The inset shows the enlarged region of the N-terminal effector lobe (residues 1–86). **b**) WT |Δϖ_N_| values are color-coded on the K-Ras·GNP·RBD complex structure (PDB 8DGT), where residues with |Δϖ_N_| > 0.8 are highlighted as spheres, and RBD is shown in pale green. The cyan sphere corresponds to Mg^2+^. **c, d**) Comparison of signed Δϖ_N_ values with the differences in ^15^N chemical shift between the RBD-bound K-Ras·GTP (Ω^K‑Ras·GTP·RBD^) and free K-Ras·GDP (Ω^K‑Ras·GDP^) for residues 1–86. WT is displayed in panel **c** and G12D in panel **d**. Residues in the P-loop, Switch I, and Switch II regions are highlighted in yellow, red, and blue, respectively. The root-mean-squared-deviation (RMSD) is 1.37 ppm for WT effector lobe and 1.31 ppm for G12D with a Pearson correlation coefficient R of 0.89 for WT and 0.91 for G12D. **e**) Correlation plot between the ^15^N chemical shift of the lowly populated RBD-bound K-Ras·GDP state (*y*-axis) with the RBD-bound K-Ras·GTP state (*x*-axis) for all residues. Errors in Δϖ_N_ values represent 1 s.d. and are derived from 500 bootstrap simulations. The error bars in panels **c**, **d**, and **e** are too small to be visually discerned.

To evaluate whether the RBD-bound K-Ras·GDP state corresponds to a similar structural end point as the active RBD-bound K-Ras·GTP complex, we compared the signed Δϖ values with the independently determined chemical shift differences between the RBD-bound K-Ras·GTP (Ω^K‑Ras·GTP·RBD^) and free K-Ras·GDP (Ω^K‑Ras·GDP^). For both WT and G12D, the Δϖ and Ω^K‑Ras·GTP·RBD^ – Ω^K‑Ras·GDP^ values agree well, with high Pearson correlation coefficients *R* of 0.89 (WT) and 0.91 (G12D) for the effector lobe (Figure 4c, d). The close agreement suggests that the transient RBD-bound K-Ras·GDP state adopts a conformation that closely matches that of the active RBD-bound K-Ras·GTP state. This can be clearly seen from the high correlation between the ^15^N chemical shifts of RBD-bound K-Ras·GDP and the RBD-bound K-Ras·GTP state (Figure 4e) with Pearson *R* of 0.98 (WT) and 0.99 (G12D), and chemical shift RMSDs of 1.00 ppm (WT) and 0.95 ppm (G12D) for all residues. The largest deviations from the diagonal occur for residues G13 and G60, both of which lie in close proximity to the γ-phosphate of GTP. These deviations are likely dominated by differences in the local chemical environment between GTP and GDP.

### RBD Interactions with K-Ras·GDP

We carried out reciprocal experiments in which ^15^N-labeled B-Raf RBD was monitored while unlabeled K-Ras·GDP was added to obtain a complementary assessment of the interaction from the effector perspective. As for the K-Ras-observed data sets, backbone ^1^H–^15^N HSQC spectra of RBD show subtle chemical shift perturbations and concentration-dependent line broadening for a subset of cross peaks (Figure S4) upon addition of equimolar (or less) unlabeled K-Ras·GDP. This transient interaction between RBD and K-Ras·GDP was quantified with ^15^N CEST and CPMG experiments with optimized protein concentrations shown in Table 1. Again, a subset of residues displayed clear CPMG dispersion, characterized by elevated effective *R*_2_ at low CPMG frequencies and a decrease in *R*_2_ when increasing CPMG frequencies. Those residues also exhibited secondary saturation dips in the CEST profile with representative examples shown in Figures 5 and S5. Again, the CEST and CPMG profiles can be explained by a two-state exchange process consistent with the transient interaction between RBD and K-Ras·GDP. As a control, free RBD alone shows no detectable conformational exchange on the millisecond time scale with no dispersion in CPMG profiles and no evidence of secondary saturation dips in CEST profiles (Figure S6).

**Figure 5:**
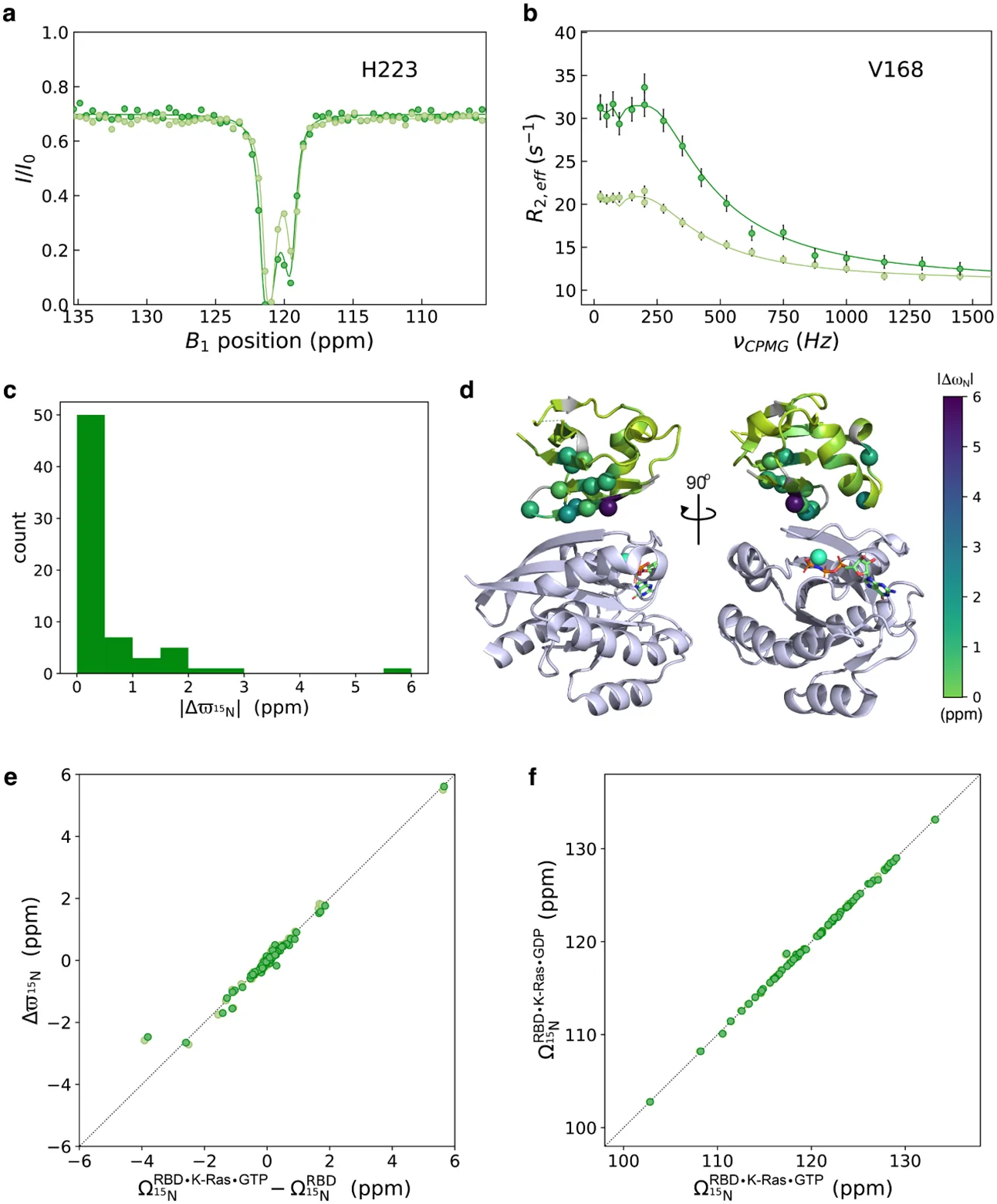
Chemical shifts of RBD in the K-Ras·GDP-bound state derived by CEST and CPMG. Representative **a**) ^15^N CEST and **b**) ^15^N CPMG profiles of ^15^N-labeled RBD in the presence of unlabeled WT (green) and G12D (light green) K-Ras·GDP, demonstrating the transient interaction between RBD and K-Ras·GDP. Additional profiles are shown in the Supporting Information (Figure S5). **c**) The distribution of absolute ^15^N chemical shift differences |Δϖ_N_| of RBD between its free and WT K-Ras·GDP-bound states derived from ^15^N CEST and CPMG experiments. **d**) Mapping of the RBD |Δϖ_N_| values of **c**) on the K-Ras·GNP·RBD complex structure (PDB 8DGT), where residues with |Δϖ_N_| > 0.8 are highlighted as green to blue spheres, and K-Ras is shown in light blue. The cyan sphere in K-Ras·GNP corresponds to Mg^2+^. **e**) Comparison of signed Δϖ_N_ values with the differences in ^15^N chemical shift between the K-Ras·GTP-bound RBD (Ω^RBD·K‑Ras·GTP^) and free RBD (Ω^RBD^). The RMSD is 0.20 ppm for RBD interacting with WT K-Ras (green) and 0.19 ppm for RBD interacting with G12D K-Ras (light green) with a Pearson correlation coefficient R of 0.98 for both WT and G12D. **f**) The ^15^N chemical shifts are highly correlated between the lowly populated K-Ras·GDP-bound RBD and K-Ras·GTP-bound RBD.

The extracted binding parameters are summarized in Table 1. The binding affinities lie in the low mM range (3.86 mM for RBD with WT K-Ras·GDP and 1.83 mM for RBD with G12D mutant) and residence times are on the ms scale (6.82 ms for WT and 12.41 ms for G12D), which are on par with the values obtained from the K-Ras·GDP side. As for site-specific ^15^N chemical shift differences Δϖ between the free and bound RBD, the largest changes are observed for residues R158, V159, K164, R166, V168, R188, I191, and H223, which are located in the β_1_, β_2_, and β_5_ strands and the end of the α-helix (Figures 5 and S7). The chemical shifts of the K-Ras·GDP-bound state of RBD show remarkable agreement with those of RBD bound to K-Ras·GTP, with Pearson correlation coefficients *R* of 0.999 for both WT and G12D and low RMSDs of 0.20 ppm (WT) and 0.19 ppm (G12D) (Figure 5f). Together, these data indicate that RBD interacts with K-Ras·GDP with the same contact interface as K-Ras·GTP while adopting a conformation closely matching that in the K-Ras·GTP-bound complex.

## Discussion

The Ras-Raf interaction is a central effector-binding event in MAPK signaling. Ras·GTP-Raf engagement is often described as high-affinity with a dissociation constant of low-nM to tens-of-nM.^14,16,53^ For B-Raf RBD and K-Ras·GNP, *K*_D_ has been reported to be 26 nM.^16^ Consistent with the high binding affinity, our HSQC experiments on K-Ras·GTP·B-Raf-RBD confirm the formation of a stable complex with a well-defined structure. RBDs of the Raf family share conserved structural features and adopt a ubiquitin-like fold.^54,56^ A high-resolution K-Ras·GNP·C-Raf-RBD X-ray crystal structure has been solved at 1.4 Å,^14^ and more recently, a cryo-EM structure of the K-Ras·GNP·B-Raf recruitment complex with MEK1 and 14–3–3 chaperone has become available at a lower resolution of 3.9 Å.^16^ According to those structures, RBD binds primarily to Switch I and the adjacent β_2_-strand of K-Ras, with the β_2_-strand of RBD forming an intermolecular antiparallel β-sheet with the β_2_-strand of K-Ras at the interface. Consistent with the existing structures, the binding-induced CSPs observed in K-Ras·GTP and RBD predominantly cluster in the effector lobe of K-Ras and the β_2_-strand and α-helix of RBD. When mapped onto the cryo-EM structure (PDB 8DGT), the residues with the largest CSPs are located directly at the contact interface in the complex (Figure S1). Besides shape complementarity, electrostatic complementarity is a major contributor to the interaction. Acidic residues in and around the K-Ras Switch I region can form hydrogen bonds and salt bridges with basic residues on the RBD β_2_-strand and α-helix, such as D33 of K-Ras with K183 of RBD and E37 of K-Ras with R166 of RBD, contributing to high-affinity binding in the nanomolar regime.

Free K-Ras·GTP interconverts between two conformational states on the millisecond time scale, in which the excited state resembles K-Ras·GDP with highly flexible Switch regions, whereas the dominant ground state is competent for effector binding.^8^ CPMG relaxation dispersion experiments indicate binding with RBD effectively quenches the two-state conformational exchange observed for free K-Ras·GTP, and the resulting complex is rather rigid. This behavior aligns with previous observations^21,57^ that RBD engagement stabilizes the effector-binding competent state by shifting the conformational equilibrium of K-Ras·GTP toward its ground state.

Remarkably, we found that K-Ras·GDP forms weak but detectable associations with B-Raf RBD. This is reflected in the protein concentration-dependent and residue-specific line broadening observed in ^1^H–^15^N HSQC spectra. ^15^N-CEST measurements unambiguously reveal the presence of a single bound state, as detected independently from the viewpoints of K-Ras·GDP as well as RBD. Together with ^15^N-CPMG experiments, we extracted the backbone ^15^N chemical shifts of the K-Ras·GDP·RBD complex, thereby gaining structural information on a complex that is sparsely populated and too transient in nature for other structural characterization methods. Residues with large chemical shift changes upon binding are in the Switch regions of K-Ras·GDP and in the β_2_-strand as well as C-terminal end of the α-helix in RBD, indicating that these regions define the interaction surface, possibly accompanied by binding-induced conformational rearrangement or population shifts. Moreover, for both WT and G12D K-Ras, the GDP-bound complexes display chemical shifts that correlate strongly with those of K-Ras·GTP·RBD, with low RMSDs and high correlation coefficients for both binding partners. Taken together, our data establish that the observed K-Ras·GDP and RBD interaction is *not* a random, i.e., nonspecific, encounter. Rather, the bound state closely resembles the K-Ras·GTP·RBD complex.

In addition to the chemical shifts of the bound state, global fitting of CPMG and CEST profiles directly yields the bound population *p*_b_ and the lifetime of the complex via the dissociation rate *k*_off_. When the protein concentrations of the samples are known, one can further derive the dissociation constant *K*_D_ and the association rate *k*_on_. Independent analyses from the K-Ras- and RBD-detected data sets yield comparable values, corroborating the consistency of the measurements. At 100 mM NaCl ionic strength, the WT K-Ras·GDP and RBD interaction falls in the low mM regime, with *K*_D_ values of 1.56 and 3.86 mM from the two independent analyses, and a residence time around 6 ms. Intriguingly, the G12D mutation strengthens the interaction, approximately doubling both the affinity and the lifetime of the complex.

Since both Switch regions of free K-Ras·GDP are much more dynamic than of K-Ras·GTP,^8^ binding of K-Ras·GDP to RBD involves a conformational entropy penalty, which is likely to contribute to the low binding affinity. Weak protein–protein interactions with *K*_D_ in the μM—mM regime are increasingly recognized as functionally important, supporting molecular recognition, signaling, and dynamic assembly formation.^58–61^ However, such interactions are challenging to characterize by methods that rely on stable complex formation without introducing engineered modifications to stabilize the interaction. Solution NMR is uniquely powerful in providing structural information on sparsely populated and short-lived binding complexes under near-physiological conditions.^62,63^ CPMG relaxation dispersion and CEST experiments quantify exchange between an observable free state and a sparsely populated bound state. These methods, individually and in combination, have illuminated the coupled folding upon binding mechanism between intrinsically disordered proteins or disordered regions and globular binding partners,^64–69^ such as pKID-KIX^64^ and POSH-Rac1 ^69^ interactions. CPMG also captures weak self-association, for example the dimerization of GB1 mutant.^70^ Here, we find that such type of behavior is also possible for heterodimeric interactions involving the two globular proteins Ras·GDP and RBD. Other NMR approaches to study weak protein–protein interactions include dark state exchange saturation transfer (DEST), which allows characterization of transient binding to high molecular weight “dark” species,^71,72^ as exemplified by studies of amyloid-β peptide binding to GroEL.^73^ Paramagnetic relaxation enhancement (PRE) is particularly informative for probing ultraweak complexes and transient encounters,^74^ for example, the weak self-association of HPr^75^ and the interaction between the two enzymes EI and EIIA^Glc^ (*K*_D_ around 25 mM) that supports the bacterial phosphorylation signaling.^59^

The oncogenic G12D mutant of K-Ras exhibits a moderate enhancement of the binding between K-Ras·GDP and RBD. While the K-Ras·GNP·B-Raf structure has been solved by cryo-EM,^16^ there is no B-Raf RBD (nor any other RBD in the Raf family) with G12D K-Ras·GTP/analog structure available. Therefore, we modeled the G12D K-Ras·GNP·RBD structure by replacing residue G12 in the cryo-EM WT K-Ras·GNP·RBD structure^16^ (PDB 8DGS) with D12 with its side-chain conformation taken from the G12D K-Ras·GDP crystal structure^76^ (PDB 4EPR) (Figure S8). The resulting structure shows that the side-chain amide of Q61 is positioned near the D12 side-chain carboxylic acid, with a measured distance of 3.8 Å between the amide nitrogen and the carbonyl oxygen, suggesting likely hydrogen-bond formation. It could help stabilize G12D K-Ras·GDP in a conformation that is favorable for RBD binding, resulting in enhanced affinity between G12D K-Ras·GDP with RBD compared to WT K-Ras allowing for a longer lifetime τ_res_ of the bound state.

Previous studies have shown that GDP-state Ras-effector recognition can be enabled by mutations in either the effector or the GTPase itself. On the effector side, an engineered A85K mutation in C-Raf RBD enhances its affinity for H-Ras·GDP, yielding a *K*_D_ comparable to that reported for the interaction between H-Ras·GNP and an alternative effector RalGDS.^77^ Structural analysis by X-ray crystallography further revealed that, in the mutant complex, Switch I of H-Ras·GDP adopts a more GTP-like conformation, albeit with higher flexibility than in the GTP-bound effector complex as suggested by the crystallographic B-factors.^78^ For the Ras GTPase superfamily, the tumor-derived RhoA A161P and A161V mutants were found to interact with RBDs of downstream effectors in the GDP-bound state, and HSQC measurements indicated that A161V RhoA·GDP can sample an active, RhoA·GTP-like conformation.^79^ Similarly, in K-Ras, molecular dynamics simulations have shown that the oncogenic G12V mutant can sample the active-like conformations that resemble those of K-Ras·GTP in the GDP-bound state.^80^ More recently, G12D K-Ras·GDP was reported to retain a low-affinity interaction with B-Raf RBD.^32^

Our results demonstrate that wild-type K-Ras·GDP interacts with B-Raf RBD in a highly specific manner by adopting a complex, which structurally closely matches that of K-Ras·GTP·RBD. There is no evidence of formation of alternative complexes between K-Ras·GDP and RBD, including nonspecific interactions. With a dissociation constant several orders of magnitude larger than that of the GTP-bound complex,^16^ the affinity between K-Ras·GDP and RBD is quite weak and short-lived with a residence time around 6 ms compared to K-Ras·GTP·RBD, which has an estimated lifetime from 50–100 ms to around 1 s.^27,81^ Although at bulk cellular concentrations such a weak interaction would likely be inconsequential, in the cellular environment the population of the K-Ras·GDP·RBD complex will be significantly higher since Ras and Raf can form nanoclusters at the plasma membrane, thereby substantially increasing their local effective concentrations.^82^ In such crowded environments, even weak affinities can lead to biologically relevant binding events analogous to other weak protein–protein interactions mentioned above. Moreover, the presence of the CRD subdomain may further enhance binding interactions and prolong the lifetime of the complex.^14,27^ While such a complex would still be significantly less populated than the corresponding GTP-bound complex, the GDP-bound state might nonetheless contribute to signaling albeit at a lower level. Notably, we found that the G12D mutation strengthens the interaction between K-Ras·GDP and its effector. Furthermore, certain naturally occurring oncogenic mutants may further promote K-Ras·GDP-effector association, diminishing nucleotide-state discrimination and potentially enabling signaling by the GDP-bound state of K-Ras.

## Conclusions

Considering the very different structural-dynamic behavior of free K-Ras when bound to GDP vs GTP, it is remarkable that they adopt a structurally highly similar complex with the effector RBD target domain. It attests to the high plasticity of K-Ras·GDP undergoing a major population shift when binding to RBD, which demonstrates that the effector-bound structure is “baked” into K-Ras irrespective of the state of its nucleotide ligand. While K-Ras·GTP is known as the active state of K-Ras due to its binding competency with RBD that triggers the MAPK downstream signaling cascade, K-Ras·GDP is also able to specifically and transiently bind to RBD in its WT and even stronger in the oncogenic G12D mutant as revealed here using solution NMR experiments. These insights should assist novel therapeutic modalities targeting not only the K-Ras·GTP active state, but also specific conformational substates accessible by K-Ras·GDP including those that retain effector-binding competence.

Although we focused on K-Ras and its interactions with the B-Raf RBD domain, the general type of behavior described here may also be relevant for other allosteric protein–protein interactions where one of the proteins toggles between an active high-affinity and an inactive low-affinity state. In such systems, the active state may be inherently present at low population in the conformational ensemble of the mostly inactive state promoting constitutive activity. Such behavior could allow alternate therapeutic molecular targeting strategies by reducing the population of these active conformations, thereby blocking signaling-critical interactions with effector protein partner(s).

## Supplementary Material



## Data Availability

NMR resonance assignments of the complex of K-Ras·GTP with B-Raf RBD domain have been deposited in the BMRB database^44^ (accession codes 53732 (K-Ras) and 53733 (RBD domain)). All CEST and CPMG data of the K-Ras·GDP·RBD complex have been deposited in the Dryad repository.^85^

## Notes

Nucleotide and amino acid sequences of K-Ras and RBD; summary of resonance assignments; parameter settings used for dynamics experiments; figures of chemical shift perturbations, CPMG and CEST profiles, chemical shift differences in RBD upon K-Ras·GDP binding, and 3D structural illustration of K-Ras·GNP·RBD complex (PDF)

## References

[1] Ellis C. A., Clark G. (2000). The Importance of Being K-Ras. Cell. Signalling.

[2] McCormick F. (2022). A Brief History
of RAS and the RAS Initiative. Adv. Cancer Res..

[3] Bhattarai N., Buck M. (2026). Recent Breakthroughs
in Understanding the Allosteric Features of
Ras GTPases and Their Effector and Regulatory Protein Interactions,
Enabling Drug Design. Curr. Opin. Struct. Biol..

[4] Bos J. L., Rehmann H., Wittinghofer A. (2007). GEFs and GAPs:
Critical Elements
in the Control of Small G Proteins. Cell.

[5] Hennig A., Markwart R., Esparza-Franco M. A., Ladds G., Rubio I. (2015). Ras Activation
Revisited: Role of GEF and GAP Systems. Biol.
Chem..

[6] Prior I. A., Hood F. E., Hartley J. L. (2020). The Frequency of Ras Mutations in
Cancer. Cancer Res..

[7] Pantsar T. (2020). The Current
Understanding of KRAS Protein Structure and Dynamics. Comput. Struct. Biotechnol. J..

[8] Hansen A. L., Xiang X., Yuan C., Bruschweiler-Li L., Brüschweiler R. (2023). Excited-State Observation of Active
K-Ras Reveals Differential
Structural Dynamics of Wild-Type versus Oncogenic G12D and G12C Mutants. Nat. Struct. Mol. Biol..

[9] Hillig R. C., Sautier B., Schroeder J., Moosmayer D., Hilpmann A., Stegmann C. M., Werbeck N. D., Briem H., Boemer U., Weiske J., Badock V., Mastouri J., Petersen K., Siemeister G., Kahmann J. D., Wegener D., Böhnke N., Eis K., Graham K., Wortmann L., Von Nussbaum F., Bader B. (2019). Discovery of Potent SOS1 Inhibitors
That Block RAS Activation via Disruption of the RAS–SOS1 Interaction. Proc. Natl. Acad. Sci. U. S. A..

[10] Rabara D., Tran T. H., Dharmaiah S., Stephens R. M., McCormick F., Simanshu D. K., Holderfield M. (2019). KRAS G13D
Sensitivity to Neurofibromin-Mediated
GTP Hydrolysis. Proc. Natl. Acad. Sci. U. S.
A..

[11] Yan W., Markegard E., Dharmaiah S., Urisman A., Drew M., Esposito D., Scheffzek K., Nissley D. V., McCormick F., Simanshu D. K. (2020). Structural Insights into the SPRED1-Neurofibromin-KRAS
Complex and Disruption of SPRED1-Neurofibromin Interaction by Oncogenic
EGFR. Cell Rep..

[12] Castel P., Dharmaiah S., Sale M. J., Messing S., Rizzuto G., Cuevas-Navarro A., Cheng A., Trnka M. J., Urisman A., Esposito D., Simanshu D. K., McCormick F. (2021). RAS Interaction
with Sin1 Is Dispensable for mTORC2 Assembly and Activity. Proc. Natl. Acad. Sci. U. S. A..

[13] Moghadamchargari Z., Shirzadeh M., Liu C., Schrecke S., Packianathan C., Russell D. H., Zhao M., Laganowsky A. (2021). Molecular
Assemblies of the Catalytic Domain of SOS with KRas and Oncogenic
Mutants. Proc. Natl. Acad. Sci. U. S. A..

[14] Tran T. H., Chan A. H., Young L. C., Bindu L., Neale C., Messing S., Dharmaiah S., Taylor T., Denson J.-P., Esposito D., Nissley D. V., Stephen A. G., McCormick F., Simanshu D. K. (2021). KRAS Interaction with RAF1 RAS-Binding Domain and Cysteine-Rich
Domain Provides Insights into RAS-Mediated RAF Activation. Nat. Commun..

[15] Eves B. J., Gebregiworgis T., Gasmi-Seabrook G. M.
C., Kuntz D. A., Privé G. G., Marshall C. B., Ikura M. (2022). Structures of RGL1
RAS-Association Domain in Complex with KRAS and the Oncogenic G12V
Mutant. J. Mol. Biol..

[16] Park E., Rawson S., Schmoker A., Kim B.-W., Oh S., Song K., Jeon H., Eck M. J. (2023). Cryo-EM Structure
of a RAS/RAF Recruitment Complex. Nat. Commun..

[17] Li B., Wang H., Wang H., Fang M., Long D. (2025). Dynamic Binding
of HRas·GTP to the Allosteric Site of Sos Investigated by Solution
NMR. JUSTC.

[18] Yuan C., Hansen A. L., Bruschweiler-Li L., Brüschweiler R. (2024). NMR ^1^H, ^13^C, ^15^N Backbone
Resonance Assignments
of Wild-Type Human K-Ras and Its Oncogenic Mutants G12D and G12C Bound
to GTP. Biomol. NMR Assign..

[19] Xu S., Long B. N., Boris G. H., Chen A., Ni S., Kennedy M. A. (2017). Structural Insight
into the Rearrangement of the Switch
I Region in GTP-Bound G12A K-Ras. Acta Crystallogr.:
D Struct Biol..

[20] Menyhárd D. K., Pálfy G., Orgován Z., Vida I., Keserű G. M., Perczel A. (2020). Structural Impact of GTP Binding on Downstream KRAS
Signaling. Chem. Sci..

[21] Geyer M., Schweins T., Herrmann C., Prisner T., Wittinghofer A., Kalbitzer H. R. (1996). Conformational
Transitions in p21*
^ras^
* and in Its Complexes
with the Effector Protein Raf-RBD
and the GTPase Activating Protein GAP. Biochemistry.

[22] Spoerner M., Herrmann C., Vetter I. R., Kalbitzer H. R., Wittinghofer A. (2001). Dynamic Properties of the Ras Switch
I Region and Its
Importance for Binding to Effectors. Proc. Natl.
Acad. Sci. U. S. A..

[23] Parker J. A., Volmar A. Y., Pavlopoulos S., Mattos C. (2018). K-Ras Populates Conformational
States Differently from Its Isoform H-Ras and Oncogenic Mutant K-RasG12D. Structure.

[24] Downward J. (2003). Targeting
RAS Signalling Pathways in Cancer Therapy. Nat.
Rev. Cancer.

[25] Mozzarelli A. M., Simanshu D. K., Castel P. (2024). Functional and Structural Insights
into RAS Effector Proteins. Mol. Cell.

[26] Wellbrock C., Karasarides M., Marais R. (2004). The RAF Proteins Take Centre Stage. Nat. Rev. Mol. Cell Biol..

[27] Jimenez
Salinas A., Tevdorashvili K., Grim J., Morales A., Chakhrakia A., Kwang Lee Y. (2026). Positive Cooperativity between RAS-Binding
and Cysteine-Rich Domains Regulates RAF Membrane Binding Kinetics
via Lateral Rebinding. Nat. Commun..

[28] Terrell E. M., Morrison D. K. (2019). Ras-Mediated Activation
of the Raf Family Kinases. Cold Spring Harbor
Perspect. Med..

[29] Park E., Rawson S., Li K., Kim B.-W., Ficarro S. B., Pino G. G.-D., Sharif H., Marto J. A., Jeon H., Eck M. J. (2019). Architecture of Autoinhibited and Active BRAF–MEK1–14-3-3
Complexes. Nature.

[30] Strakhova, R. ; Smith, M. J. Profiling Complex RAS-Effector Interactions Using NMR Spectroscopy. In KRAS: Methods and Protocols, Stephen, A. G. ; Esposito, D. Eds.; Springer: US, New York, NY, 2024; pp. 195–209. 10.1007/978-1-0716-3822-4_14. 38570461

[31] Rennella E., Henry C., Dickson C. J., Georgescauld F., Wales T. E., Erdmann D., Cotesta S., Maira M., Sedrani R., Brachmann S. M., Ostermann N., Engen J. R., Kay L. E., Beyer K. S., Wilcken R., Jahnke W. (2025). Dynamic Conformational Equilibria in the Active States
of KRAS and NRAS. RSC Chem. Biol..

[32] Kim H.-N., Gasmi-Seabrook G. M. C., Uchida A., Gebregiworgis T., Marshall C. B., Ikura M. (2025). Switch II
Pocket Inhibitor Allosterically
Freezes KRAS^G12D^ Nucleotide-Binding Site and Arrests the
GTPase Cycle. J. Mol. Biol..

[33] Showalter S. A., Bruschweiler-Li L., Johnson E., Zhang F., Brüschweiler R. (2008). Quantitative
Lid Dynamics of MDM2 Reveals Differential Ligand Binding Modes of
the p53-Binding Cleft. J. Am. Chem. Soc..

[34] Sattler M., Schleucher J., Griesinger C. (1999). Heteronuclear Multidimensional NMR
Experiments for the Structure Determination of Proteins in Solution
Employing Pulsed Field Gradients. Prog. Nucl.
Magn. Reson. Spectrosc..

[35] Palmer A. G., Cavanagh J., Wright P. E., Rance M. (1991). Sensitivity Improvement
in Proton-Detected Two-Dimensional Heteronuclear Correlation NMR Spectroscopy. J. Magn. Reson..

[36] Schleucher J., Schwendinger M., Sattler M., Schmidt P., Schedletzky O., Glaser S. J., Sørensen O. W., Griesinger C. (1994). A General
Enhancement Scheme in Heteronuclear Multidimensional NMR Employing
Pulsed Field Gradients. J. Biomol. NMR.

[37] Grzesiek S., Bax A. (1993). Amino Acid Type Determination
in the Sequential Assignment Procedure
of Uniformly ^13^C/^15^N-Enriched Proteins. J. Biomol. NMR.

[38] Hyberts S. G., Takeuchi K., Wagner G. (2010). Poisson-Gap
Sampling and Forward
Maximum Entropy Reconstruction for Enhancing the Resolution and Sensitivity
of Protein NMR Data. J. Am. Chem. Soc..

[39] Diercks T., Coles M., Kessler H. (1999). An Efficient
Strategy for Assignment
of Cross-Peaks in 3D Heteronuclear NOESY Experiments. J. Biomol. NMR.

[40] Delaglio F., Grzesiek S., Vuister G. W., Zhu G., Pfeifer J., Bax A. (1995). NMRPipe: A Multidimensional Spectral Processing System Based on UNIX
Pipes. J. Biomol. NMR.

[41] Ying J., Delaglio F., Torchia D. A., Bax A. (2017). Sparse Multidimensional
Iterative Lineshape-Enhanced (SMILE) Reconstruction of Both Non-Uniformly
Sampled and Conventional NMR Data. J. Biomol.
NMR.

[42] Johnson B. A., Blevins R. A. (1994). NMR View: A Computer Program for
the Visualization
and Analysis of NMR Data. J. Biomol. NMR.

[43] Maciejewski M. W., Schuyler A. D., Gryk M. R., Moraru I. I., Romero P. R., Ulrich E. L., Eghbalnia H. R., Livny M., Delaglio F., Hoch J. C. (2017). NMRbox:
A Resource for Biomolecular NMR Computation. Biophys. J..

[44] Ulrich E. L., Akutsu H., Doreleijers J. F., Harano Y., Ioannidis Y. E., Lin J., Livny M., Mading S., Maziuk D., Miller Z. (2008). BioMagResBank. Nucleic Acids Res..

[45] Jiang B., Yu B., Zhang X., Liu M., Yang D. (2015). A ^15^N CPMG
Relaxation Dispersion Experiment More Resistant to Resonance Offset
and Pulse Imperfection. J. Magn. Reson..

[46] Vallurupalli P., Bouvignies G., Kay L. E. (2012). Studying “Invisible”
Excited Protein States in Slow Exchange with a Major State Conformation. J. Am. Chem. Soc..

[47] Skrynnikov N. R., Dahlquist F. W., Kay L. E. (2002). Reconstructing NMR Spectra of “Invisible”
Excited Protein States Using HSQC and HMQC Experiments. J. Am. Chem. Soc..

[48] Guenneugues M., Berthault P., Desvaux H. (1999). A Method for Determining B_1_ Field Inhomogeneity.
Are the Biases Assumed in Heteronuclear Relaxation
Experiments Usually Underestimated?. J. Magn.
Reson..

[49] Lee W., Tonelli M., Markley J. L. (2015). NMRFAM-SPARKY: Enhanced Software
for Biomolecular NMR Spectroscopy. Bioinformatics.

[50] Lee W., Rahimi M., Lee Y., Chiu A. (2021). POKY: A Software Suite
for Multidimensional NMR and 3D Structure Calculation of Biomolecules. Bioinformatics.

[51] Li D.-W., Leggett A., Bruschweiler-Li L., Brüschweiler R. (2022). COLMARq: A
Web Server for 2D NMR Peak Picking and Quantitative Comparative Analysis
of Cohorts of Metabolomics Samples. Anal. Chem..

[52] Mak J. Y. W. (2022). Determination
of Sample Concentrations by PULCON NMR Spectroscopy. Aust. J. Chem..

[53] Fischer A., Hekman M., Kuhlmann J., Rubio I., Wiese S., Rapp U. R. (2007). B- and C-RAF Display
Essential Differences in Their
Binding to Ras. J. Biol. Chem..

[54] Athuluri-Divakar S. K., Vasquez-Del Carpio R., Dutta K., Baker S. J., Cosenza S. C., Basu I., Gupta Y. K., Reddy M. V. R., Ueno L., Hart J. R., Vogt P. K., Mulholland D., Guha C., Aggarwal A. K., Reddy E. P. (2016). A Small Molecule
RAS-Mimetic Disrupts RAS Association with Effector Proteins to Block
Signaling. Cell.

[55] Palmer A. G., Kroenke C. D., Patrick
Loria J. (2001). Nuclear Magnetic Resonance Methods
for Quantifying Microsecond-to-Millisecond Motions in Biological Macromolecules. Methods Enzymol..

[56] Emerson S. D., Madison V. S., Palermo R. E., Waugh D. S., Scheffler J. E., Tsao K.-L., Kiefer S. E., Liu S. P., Fry D. C. (1995). Solution
Structure of the Ras-Binding Domain of c-Raf-1 and Identification
of Its Ras Interaction Surface. Biochemistry.

[57] Chao F.-A., Dharmaiah S., Taylor T., Messing S., Gillette W., Esposito D., Nissley D. V., McCormick F., Byrd R. A., Simanshu D. K., Cornilescu G. (2022). Insights into
the Cross Talk between Effector and Allosteric Lobes of KRAS from
Methyl Conformational Dynamics. J. Am. Chem.
Soc..

[58] Perkins J. R., Diboun I., Dessailly B. H., Lees J. G., Orengo C. (2010). Transient
Protein-Protein Interactions: Structural, Functional, and Network
Properties. Structure.

[59] Xing Q., Huang P., Yang J., Sun J., Gong Z., Dong X., Guo D., Chen S., Yang Y., Wang Y., Yang M., Yi M., Ding Y., Liu M., Zhang W., Tang C. (2014). Visualizing
an Ultra–Weak
Protein–Protein Interaction in Phosphorylation Signaling. Angew. Chem., Int. Ed..

[60] Van
De Water K., Sterckx Y. G. J., Volkov A. N. (2015). The Low-Affinity
Complex of Cytochrome c and Its Peroxidase. Nat. Commun..

[61] Chen L., Cheng X., Zhang F., Dong X., Wang X., Jiang W., Ma L., Xing Q. (2026). Ultraweak Interactions
Drive Cap-Mediated Positioning and Elongation of the Bacterial Flagellar
Filament. Nat. Commun..

[62] Vaynberg J., Qin J. (2006). Weak Protein–Protein
Interactions as Probed by NMR Spectroscopy. Trends Biotechnol..

[63] Liu Z., Gong Z., Dong X., Tang C. (2016). Transient Protein–Protein
Interactions Visualized by Solution NMR. Biochim.
Biophys. Acta, Proteins Proteomics.

[64] Sugase K., Dyson H. J., Wright P. E. (2007). Mechanism
of Coupled Folding and
Binding of an Intrinsically Disordered Protein. Nature.

[65] Sugase K., Lansing J. C., Dyson H. J., Wright P. E. (2007). Tailoring Relaxation
Dispersion Experiments for Fast-Associating Protein Complexes. J. Am. Chem. Soc..

[66] Schneider R., Maurin D., Communie G., Kragelj J., Hansen D. F., Ruigrok R. W. H., Jensen M. R., Blackledge M. (2015). Visualizing
the Molecular Recognition Trajectory of an Intrinsically Disordered
Protein Using Multinuclear Relaxation Dispersion NMR. J. Am. Chem. Soc..

[67] Charlier C., Bouvignies G., Pelupessy P., Walrant A., Marquant R., Kozlov M., De Ioannes P., Bolik-Coulon N., Sagan S., Cortes P., Aggarwal A. K., Carlier L., Ferrage F. (2017). Structure and Dynamics
of an Intrinsically Disordered
Protein Region That Partially Folds upon Binding by Chemical-Exchange
NMR. J. Am. Chem. Soc..

[68] Yang, K. ; Arai, M. ; Wright, P. E. Determining Binding Kinetics of Intrinsically Disordered Proteins by NMR Spectroscopy. In Intrinsically Disordered Proteins: Methods and Protocols, Methods in Molecular Biology, Kragelund, B. B. ; Skriver, K. , Eds.; Springer: US New York, NY, 2020; pp. 663–681. 10.1007/978-1-0716-0524-0_34. PMC7605514 32696383

[69] Kjaer L. F., Ielasi F. S., Winbolt T., Delaforge E., Tengo M., Bessa L. M., Mariño Pérez L., Boeri Erba E., Bouvignies G., Palencia A., Jensen M. R. (2025). Hierarchical
Folding-upon-Binding of an Intrinsically Disordered Protein. Nat. Commun..

[70] Jee J., Ishima R., Gronenborn A. M. (2008). Characterization of Specific Protein
Association by ^15^N CPMG Relaxation Dispersion NMR: The
GB1^A34F^ Monomer–Dimer Equilibrium. J. Phys. Chem. B.

[71] Anthis N. J., Clore G. M. (2015). Visualizing Transient
Dark States by NMR Spectroscopy. Q. Rev. Biophys..

[72] Tugarinov V., Ceccon A., Clore G. M. (2022). NMR Methods for Exploring ‘Dark’
States in Ligand Binding and Protein-Protein Interactions. Prog. Nucl. Magn. Reson. Spectrosc..

[73] Libich D. S., Fawzi N. L., Ying J., Clore G. M. (2013). Probing the Transient
Dark State of Substrate Binding to GroEL by Relaxation-Based Solution
NMR. Proc. Natl. Acad. Sci. U. S. A..

[74] Clore G. M., Iwahara J. (2009). Theory, Practice and Applications of Paramagnetic Relaxation
Enhancement for the Characterization of Transient Low-Population States
of Biological Macromolecules and Their Complexes. Chem. Rev..

[75] Tang C., Ghirlando R., Clore G. M. (2008). Visualization of Transient Ultra-Weak
Protein Self-Association in Solution Using Paramagnetic Relaxation
Enhancement. J. Am. Chem. Soc..

[76] Sun Q., Burke J. P., Phan J., Burns M. C., Olejniczak E. T., Waterson A. G., Lee T., Rossanese O. W., Fesik S. W. (2012). Discovery of Small Molecules That
Bind to K-Ras and
Inhibit Sos-Mediated Activation. Angew. Chem.,
Int. Ed..

[77] Kiel C., Filchtinski D., Spoerner M., Schreiber G., Kalbitzer H. R., Herrmann C. (2009). Improved Binding of Raf to Ras·GDP
Is Correlated with Biological Activity. J. Biol.
Chem..

[78] Filchtinski D., Sharabi O., Rüppel A., Vetter I. R., Herrmann C., Shifman J. M. (2010). What Makes Ras an Efficient Molecular Switch: A Computational,
Biophysical, and Structural Study of Ras-GDP Interactions with Mutants
of Raf. J. Mol. Biol..

[79] Lin Y., Ramelot T. A., Senyuz S., Gursoy A., Jang H., Nussinov R., Keskin O., Zheng Y. (2024). Tumor-Derived RHOA
Mutants Interact with Effectors in the GDP-Bound State. Nat. Commun..

[80] Grudzien P., Jang H., Leschinsky N., Nussinov R., Gaponenko V. (2022). Conformational
Dynamics Allows Sampling of an “Active-like” State by
Oncogenic K-Ras-GDP. J. Mol. Biol..

[81] Sydor J. R., Engelhard M., Wittinghofer A., Goody R. S., Herrmann C. (1998). Transient
Kinetic Studies on the Interaction of Ras and the Ras-Binding Domain
of c-Raf-1 Reveal Rapid Equilibration of the Complex. Biochemistry.

[82] Abankwa D., Gorfe A. A., Hancock J. F. (2007). Ras Nanoclusters:
Molecular Structure
and Assembly. Semin. Cell Dev. Biol..

[83] Sehnal D., Bittrich S., Deshpande M., Svobodová R., Berka K., Bazgier V., Velankar S., Burley S. K., Koča J., Rose A. S. (2021). Mol^*^ Viewer: Modern Web
App for 3D Visualization and Analysis of Large Biomolecular Structures. Nucleic Acids Res..

[84] Goodsell D. S., Autin L., Olson A. J. (2019). Illustrate:
Software for Biomolecular
Illustration. Structure.

[85] Xiang, X. ; Basnet, M. ; Yuan, C. ; Bruschweiler-Li, L. ; Brüschweiler, R. GDP-loaded K-Ras transiently binds to effector B-Raf RBD, mirroring the structure of the active GTP-loaded complex.Dryad Digital Repository. 10.5061/dryad.fqz612k6n. PMC13523759 42677563

