# Supplementary material for: GDP-Loaded K‑Ras Transiently Binds to Effector B‑Raf RBD, Mirroring the Structure of the Active GTP-Loaded Complex
Source: J Am Chem Soc. 2026 Aug 14;148(33):35961–72. doi: 10.1021/jacs.6c09870 (PMC13523759; doi:10.1021/jacs.6c09870)
Supplement: Supplementary file 1 [file ja6c09870_si_001.pdf]

GDP-loaded K-Ras transiently binds to effector B-Raf RBD mirroring the structure of the active GTP-loaded complex

Xinyao Xiang,[1+] Mamata Basnet,[1+] Chunhua Yuan,[2] Lei Bruschweiler-Li,[1] and Rafael Brüschweiler[1,3*]

[1]Department of Chemistry and Biochemistry, The Ohio State University, Columbus, Ohio 43210, USA

[2]Campus Chemical Instrument Center, The Ohio State University, Columbus, Ohio 43210, USA

[3]Department of Biological Chemistry and Pharmacology, The Ohio State University, Columbus, Ohio 43210, USA

[+] These authors contributed equally.

[*]To whom correspondence should be addressed:

Rafael Brüschweiler, Ph.D., E-mail: bruschweiler.1@osu.edu

Content

Nucleotide and amino acid sequences of K-Ras and B-Raf RBD

Table S1: Summary of resonance assignments and quantifiable cross-peaks

Table S2: Experimental parameters for ^{15}N-CPMG relaxation dispersion and ^{15}N-CEST experiments

Figure S1: Chemical shift perturbation upon K-Ras•GTP and RBD binding

Figure S2: Representative ^{15}N-CPMG profiles of WT K-Ras•GTP in complex with RBD

Figure S3: Representative ^{15}N-CEST and ^{15}N-CPMG profiles of ^{15}N-labeled WT (blue) and G12D (purple) K-Ras•GDP in the presence of unlabeled RBD

Figure S4: ^{1}H-^{15}N HSQC of B-Raf RBD with and without equimolar unlabeled WT K-Ras•GDP present

Figure S5: Representative ^{15}N-CEST and ^{15}N-CPMG profiles of ^{15}N-labeled B-Raf RBD in the presence of unlabeled WT K-Ras•GDP and unlabeled G12D K-Ras•GDP

Figure S6: Representative ^{15}N-CEST and ^{15}N-CPMG profiles of B-Raf RBD

Figure S7: Absolute ^{15}N chemical shift differences between RBD in the K-Ras•GDP-bound state and free state derived from ^{15}N-CEST and ^{15}N-CPMG experiments

Figure S8: Depiction of cryo-EM and X-ray crystal structures of K-Ras•GNP•RBD complex and K-Ras•GDP

Nucleotide and amino acid sequences of human K-Ras4B G-domain and B-Raf RBD

WT K-Ras (aa 1-169 with an N-terminal SNA tag after TEV cleavage)

```
      1          11         21         31         41
(SNA) MTEYKLVVVG AGGVGKSALT IQLIQNHFVD EYDPTIEDSY RKQVVIDGET

      51         61         71         81         91
      CLLDILDTAG QEEYSAMRDQ YMRTGEGFLC VFAINNTKSF EDIHHYREQI

      101        111        121        131        141
      KRVKDSEDVP MVLVGNKCDL PSRTVDTKQA QDLARSYGIP FIETSAKTRQ

      151        161
      GVDDAFYTLV REIRKHKEK
```

```
(TCCAATGCA)ATGACTGAATATAAACTTGTGGTAGTTGGAGCTGGTGGCGTAGGCAAGAGTGC
CTTGACGATACAGCTAATTCAGAATCATTTTGTGGACGAATATGATCCAACAATAGAGGATTCC
TACAGGAAGCAAGTAGTAATTGATGGAGAAACCTGTCTCTTGGATATTCTCGACACAGCAGGTC
AAGAGGAGTACAGTGCAATGAGGGACCAGTACATGAGGACTGGGGAGGGCTTTCTTTGTGTATT
TGCCATAAATAATACTAAATCATTTGAAGATATTCACCATTATAGAGAACAAATTAAAAGAGTT
AAGGACTCTGAAGATGTACCTATGGTCCTAGTAGGAAATAAATGTGATTTGCCTTCTAGAACAG
TAGACACAAAACAGGCTCAGGACTTAGCAAGAAGTTATGGAATTCCTTTTATTGAAACATCAGC
AAAGACAAGACAGGGTGTTGATGATGCCTTCTATACATTAGTTCGAGAAATTCGAAAACATAAA
GAAAAG
```

G12D K-Ras

same as WT, except G12 is substituted by D12.

B-Raf RBD domain (aa 155-227 with an N-terminal SNA tag after TEV cleavage)

```
      155    161        171        181        191
(SNA) PIVRVF LPNKQRTVVP ARCGVTVRDS LKKALMMRGL IPECCAVYRI

201        211        221
QDGEKKPIGW DTDISWLTGE ELHVEVL
```

```
(TCCAATGCA)CCCATCGTGAGGGTGTTCCTGCCCAACAAGCAGAGGACCGTGGTGCCCGCCAG
GTGCGGCGTGACCGTGAGGGACAGCCTGAAGAAGGCCCTGATGATGAGGGGCCTGATCCCCGAG
TGCTGCGCCGTGTACAGGATCCAGGACGGCGAGAAGAAGCCCATCGGCTGGGACACCGACATCA
GCTGGCTGACCGGCGAGGAGCTGCACGTGGAGGTGCTG
```

Table S1. Summary of NMR resonance assignments with the number of quantifiable peaks in amide ^{15}N relaxation dispersion datasets. Apart from the three-residue SNA tag in the N-terminus from TEV cleavage during purification, K-Ras includes 169 residues numbering 1 to 169 and RBD includes 73 residues numbering 155 to 227 following convention. The alanine residue in the SNA tag of K-Ras is denoted as residue 0. Residues 34, 110, 121, and 140 of K-Ras and 155, 162, 170, 192, and 207 of RBD are prolines and do not appear in amide ^{15}N experiments.

Sample	UNASSIGNED	QUANTIFIABLE [a]
WT K-Ras•GTP in complex with unlabeled RBD	F78, T87, H94	155 / 166 (93.4%)
G12D K-Ras•GTP in complex with unlabeled RBD	F78, T87, H94[b]	--
WT K-Ras•GDP[c]	None	160 / 166 (96.4%)
G12D K-Ras•GDP[c]	None	157 / 166 (94.6 %)
RBD in complex with unlabeled WT K-Ras•GTP	N163[d]	--
RBD in complex with unlabeled G12D K-Ras•GTP	N163	--
B-Raf RBD	N163	67 / 68 (98.0%)

[a] "--" indicates no ^{15}N dynamics experiments were performed on the sample

[b] assignment transferred from WT dataset

[c] see Hansen, A. L.; Xiang, X.; Yuan, C.; Bruschweiler-Li, L.; Brüschweiler, R. Excited-state observation of active K-Ras reveals differential structural dynamics of wild-type versus oncogenic G12D and G12C mutants. *Nature Structural & Molecular Biology*, *30*, 1446-1455 (2023) (https://doi.org/10.1038/s41594-023-01070-z).

[d] assignment transferred from G12D K-Ras•GTP dataset

Table S2. Summary of parameters used in NMR ^{15}N-CPMG relaxation dispersion and ^{15}N-CEST experiments. The relaxation delays for the CPMG blocks and the calibrated B_1-field strengths and saturation times for CEST are listed. The last column indicates the number of profiles used for the global fitting of k_{ex} and p_b.

**Sample**	**^{15}N T_{CPMG}**	**HSQC/HMQC**	**^{15}N CEST B_1 / saturation time**	
B-Raf RBD	40 ms		20.8 Hz / 250 ms	
WT K-Ras•GTP in complex with unlabeled RBD	30 ms			
WT K-Ras•GDP in the presence of unlabeled RBD	40 ms	X	20.7 Hz / 150 ms	41 ^{15}N CEST<br>41 ^{15}N CPMG
G12D K-Ras•GDP in the presence of unlabeled RBD	40 ms	X	20.8 Hz / 200 ms	43 ^{15}N CEST<br>43 ^{15}N CPMG
RBD in the presence of unlabeled WT K-Ras•GDP	40 ms	X	20.8 Hz / 250 ms	29 ^{15}N CEST<br>29 ^{15}N CPMG
RBD in the presence of unlabeled G12D K-Ras•GDP	40 ms	X	20.8 Hz / 250 ms	20 ^{15}N CEST<br>20 ^{15}N CPMG

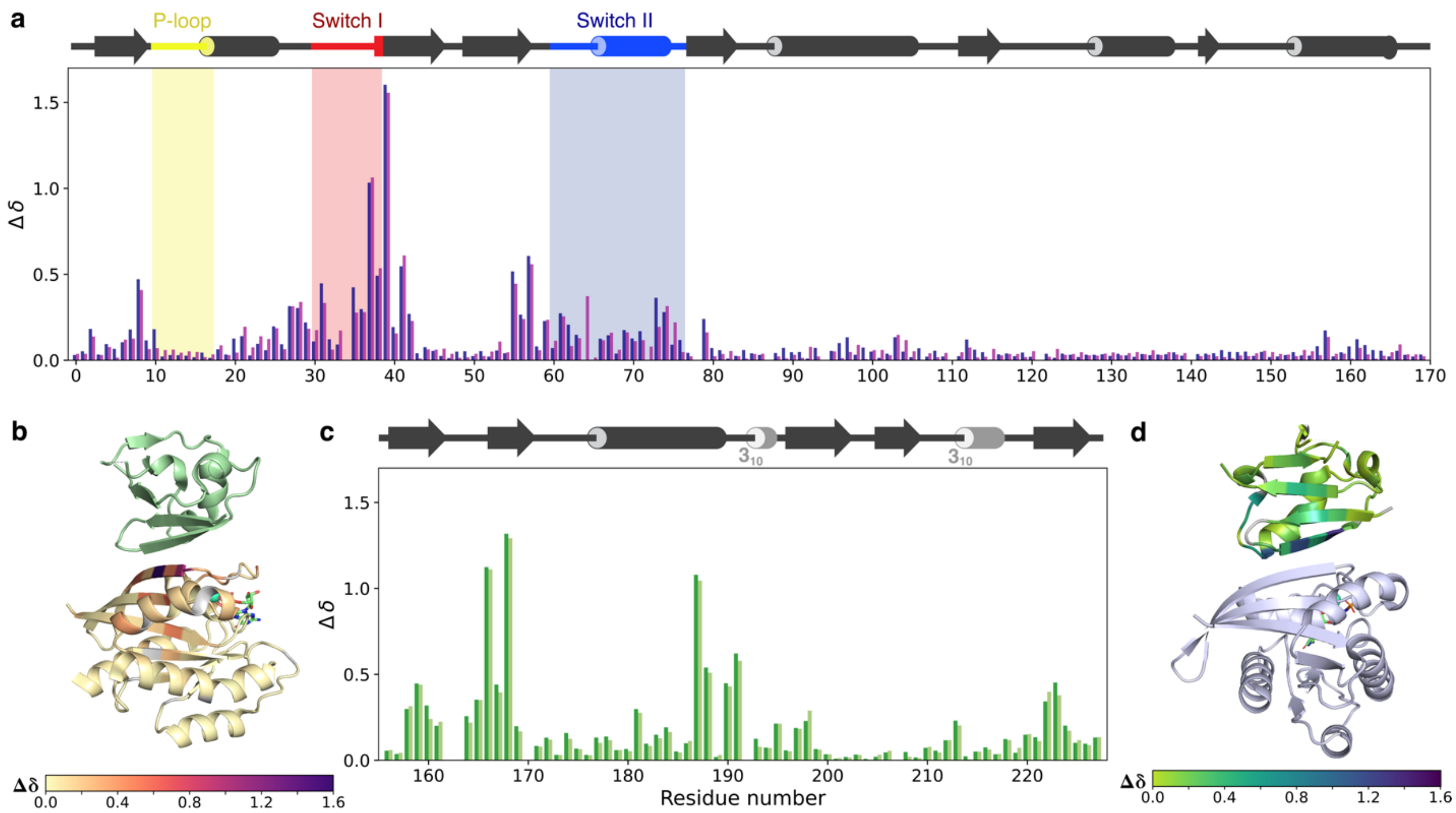

Figure S1. Chemical shift perturbation upon K-Ras•GTP and RBD binding. Changes in chemical shifts Δδ of **a**) WT (dark blue) and G12D (purple) K-Ras•GTP when interacting with equimolar RBD and **c**) B-Raf RBD when interacting with equimolar WT (green) and G12D (light green) K-Ras•GTP. $\Delta\delta = \sqrt{(\Delta\delta_N/\sigma_N)^2 + (\Delta\delta_H/\sigma_H)^2}$, where $\Delta\delta_N$ and $\Delta\delta_H$ are changes in the ^{15}N and ^{1}H chemical shifts, and σ_N = 5.218 ppm and σ_H = 0.634 ppm are the standard deviations of amide ^{15}N and ^{1}H chemical shifts used previously (Hansen, A. L. et al. *Nature Structural & Molecular Biology*, **2023**, *30*, 1446-1455). (**b,d**) Mapping of chemical shift perturbation on the cryo-EM structure of K-Ras•GNP•RBD complex (PDB 8DGT). RBD is shown in light green in panel **b** and K-Ras in light blue in panel **d**.

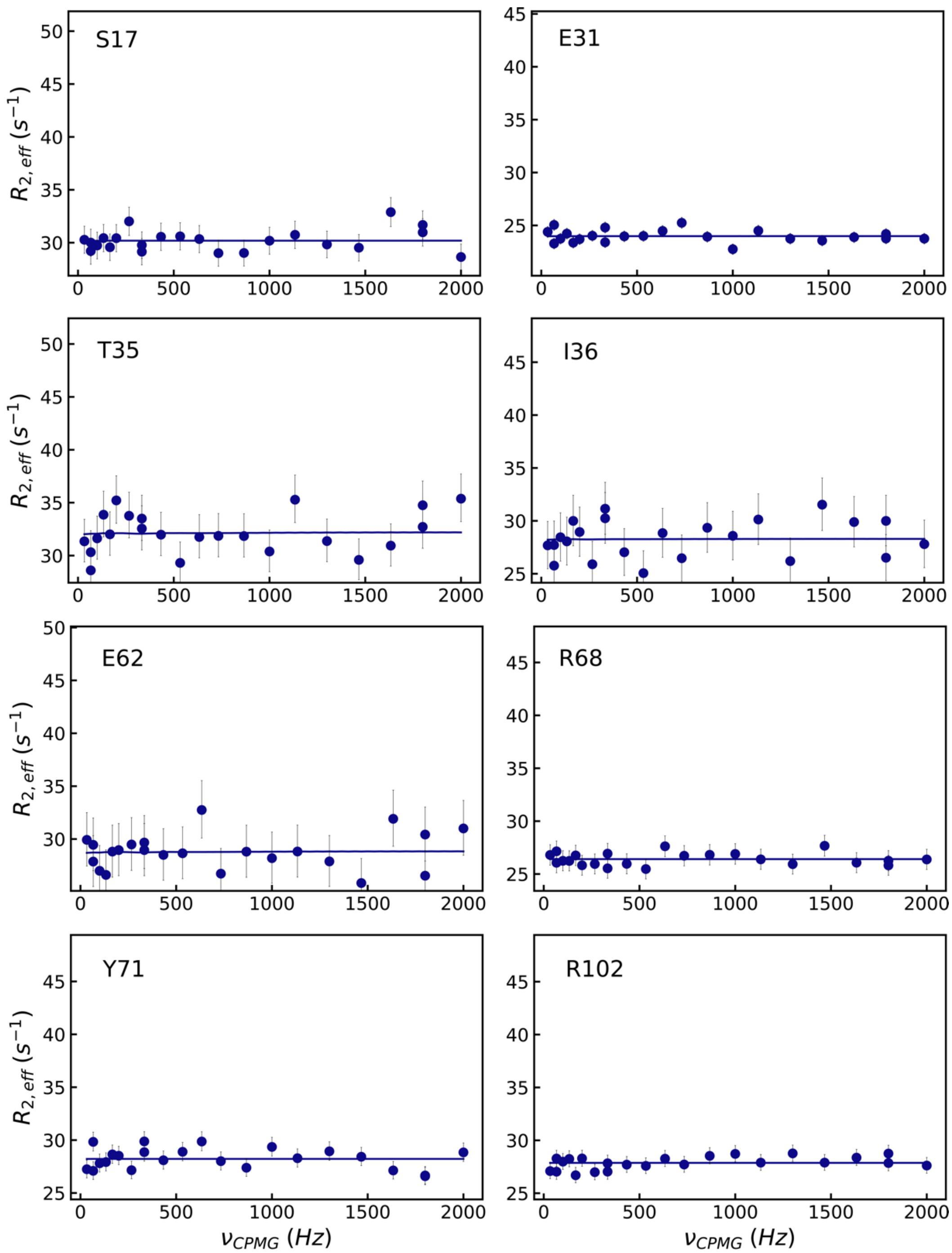

Figure S2. Representative ^{15}N-CPMG profiles of WT K-Ras•GTP in complex with RBD. The profiles indicate that millisecond conformational exchange previously observed in free K-Ras•GTP is quenched when bound with RBD.

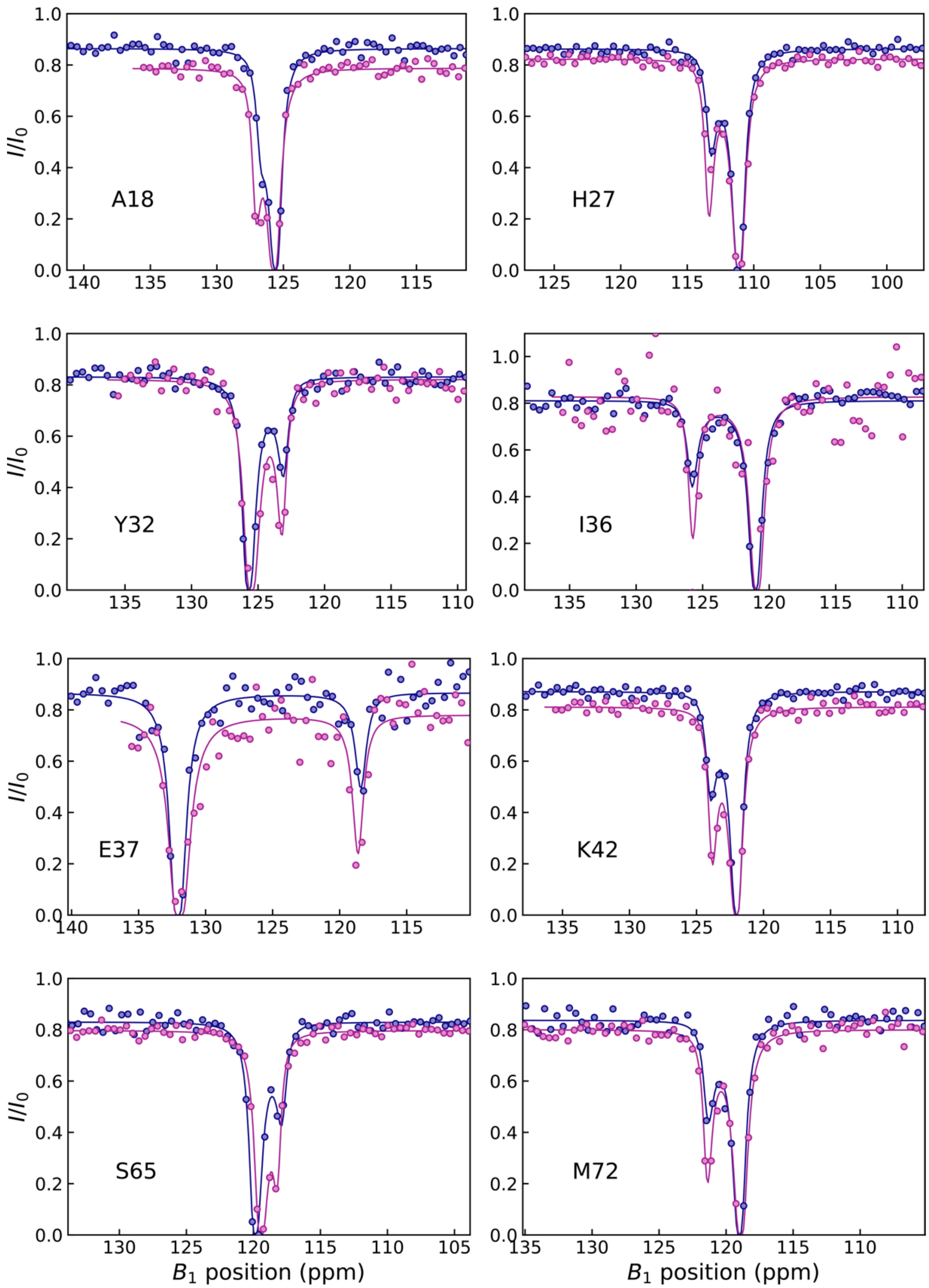
A18
H27
Y32
I36
E37
K42
S65
M72
I/I_0
B_1 position (ppm)

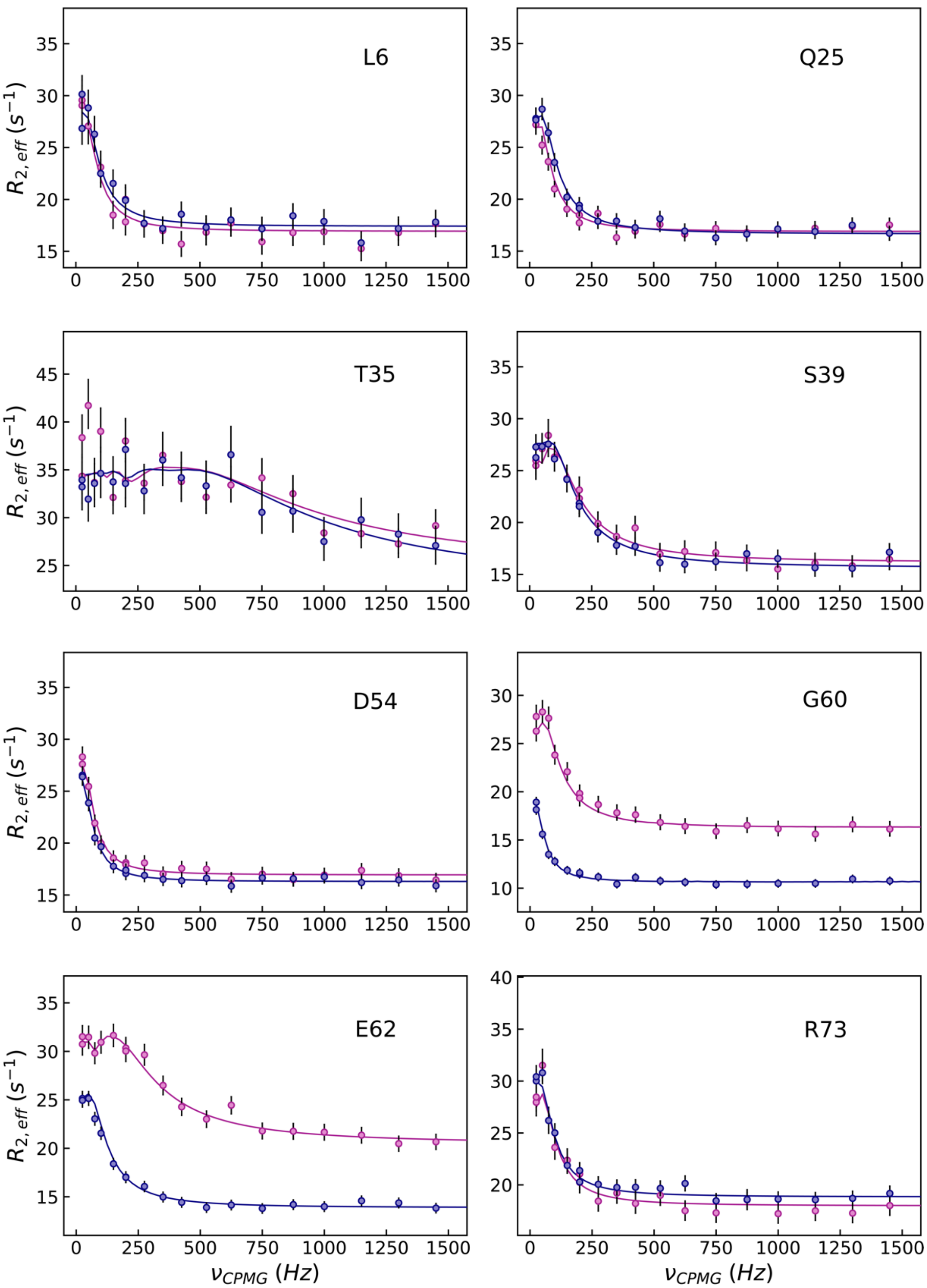

Figure S3. Representative ^{15}N-CEST and ^{15}N-CPMG profiles of ^{15}N-labeled WT (blue) and G12D (purple) K-Ras•GDP in the presence of (unlabeled) RBD.

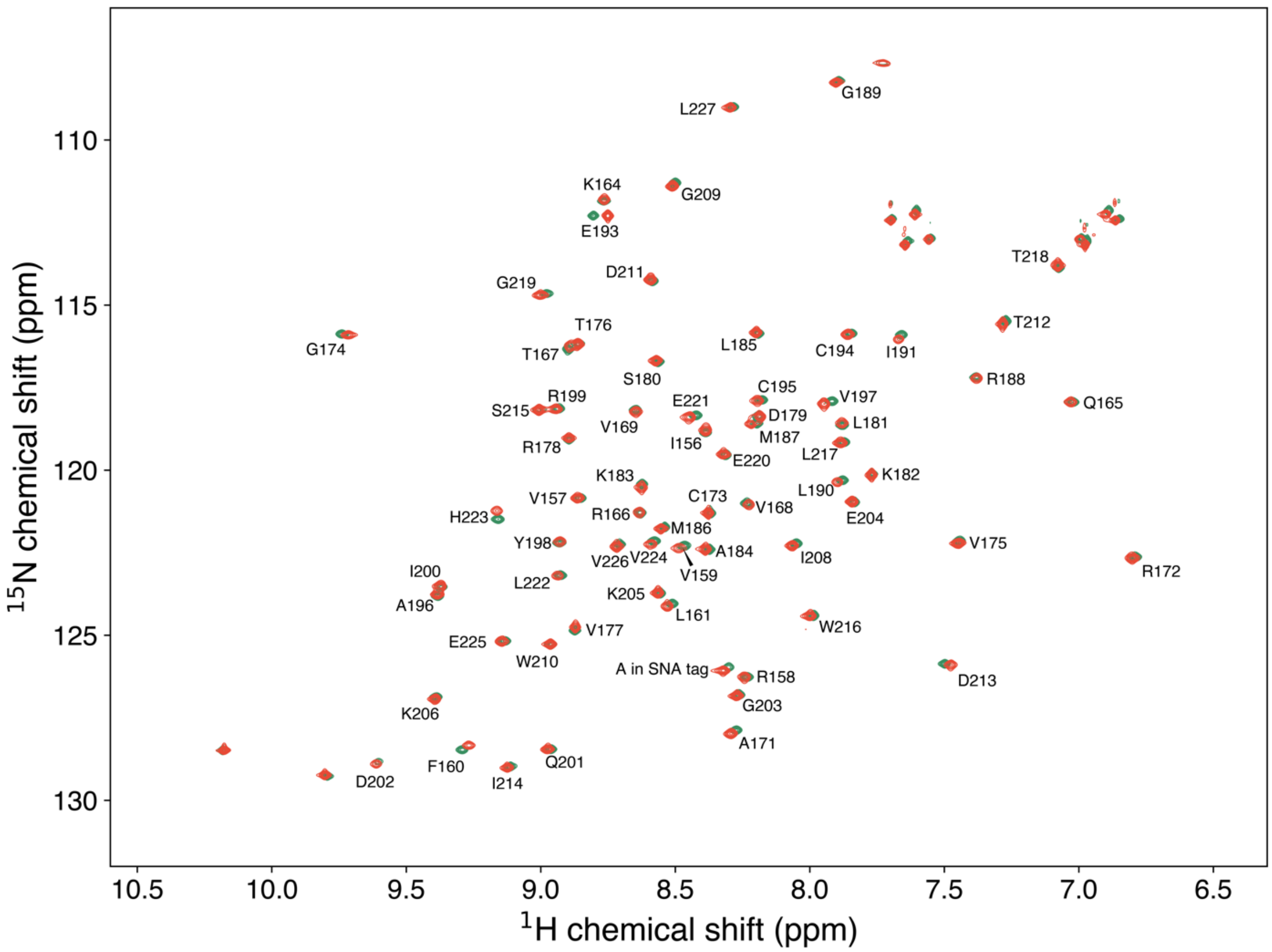

Figure S4. ^{1}H-^{15}N HSQC of B-Raf RBD without (green) and with (coral) equimolar unlabeled WT K-Ras•GDP present with backbone amide assignments indicated next to the cross-peaks. The presence of K-Ras•GDP induces relatively subtle, but clearly discernible chemical shift changes.

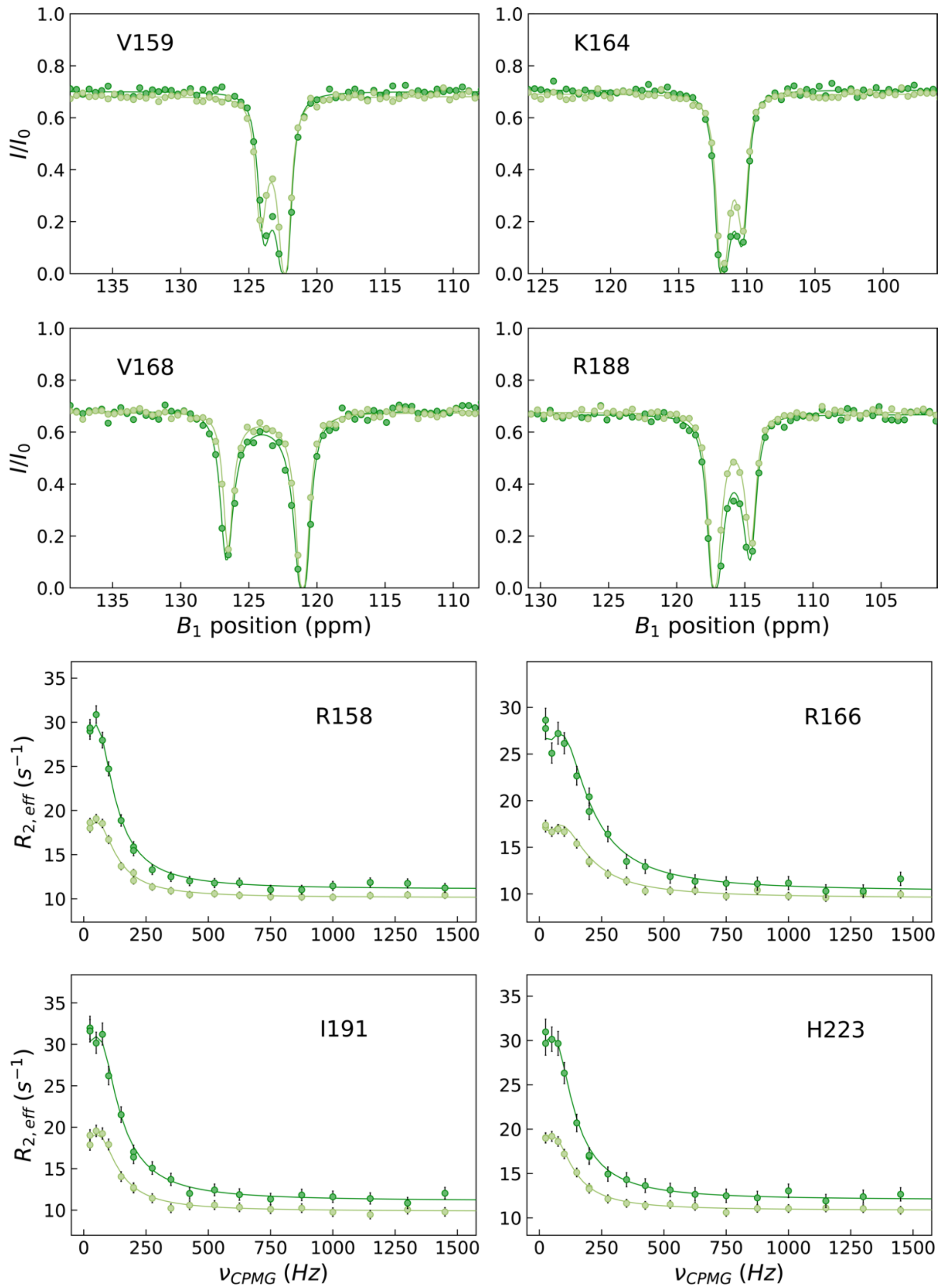

Figure S5. Representative ^{15}N-CEST and ^{15}N-CPMG profiles of ^{15}N-labeled B-Raf RBD in the presence of unlabeled WT K-Ras•GDP (green) and unlabeled G12D K-Ras•GDP (light green).

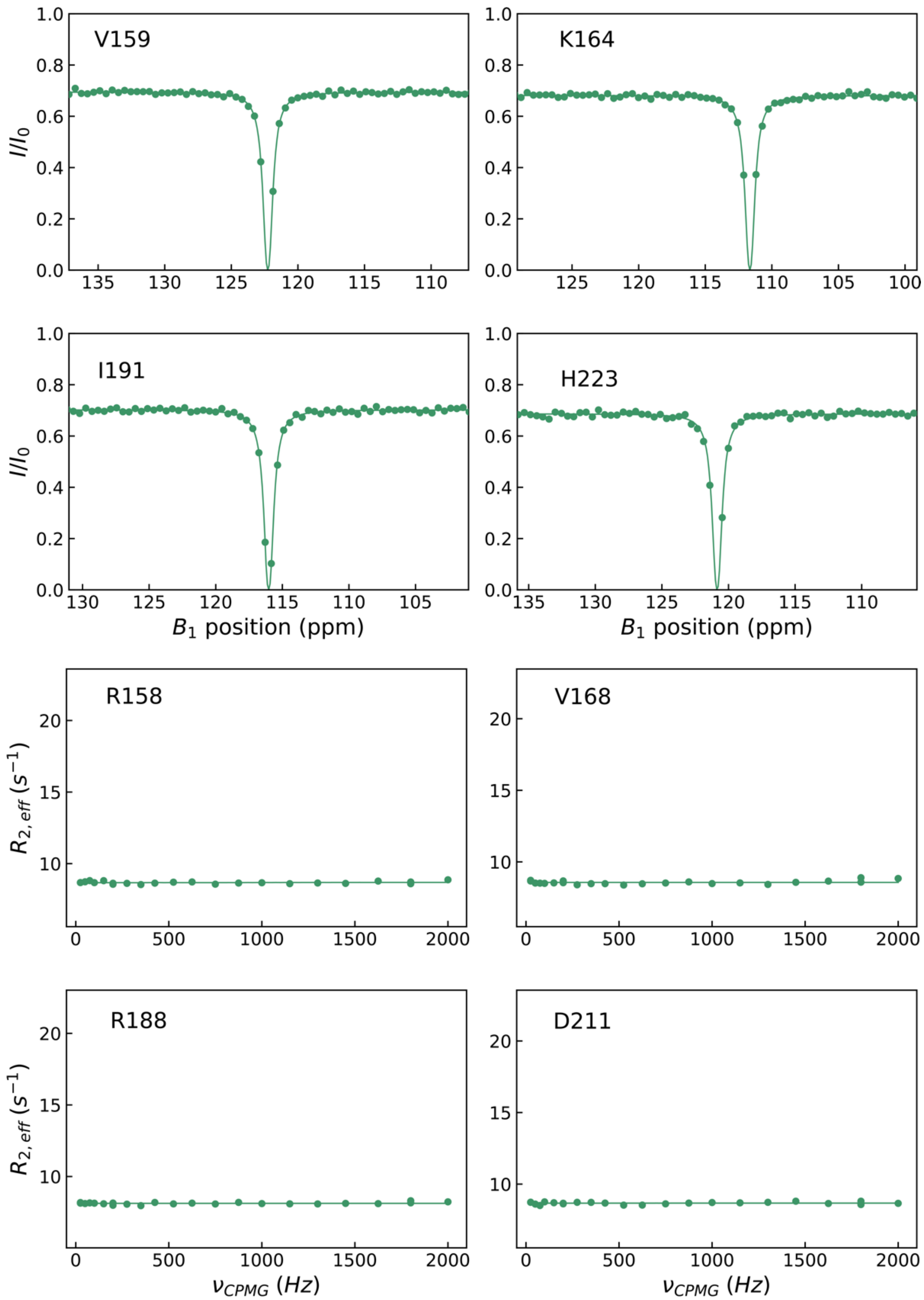

Figure S6. Representative ^{15}N-CEST and ^{15}N-CPMG profiles of B-Raf RBD. The backbone of free RBD is relatively rigid with no evidence of millisecond conformational exchange according to CEST and CPMG profiles.

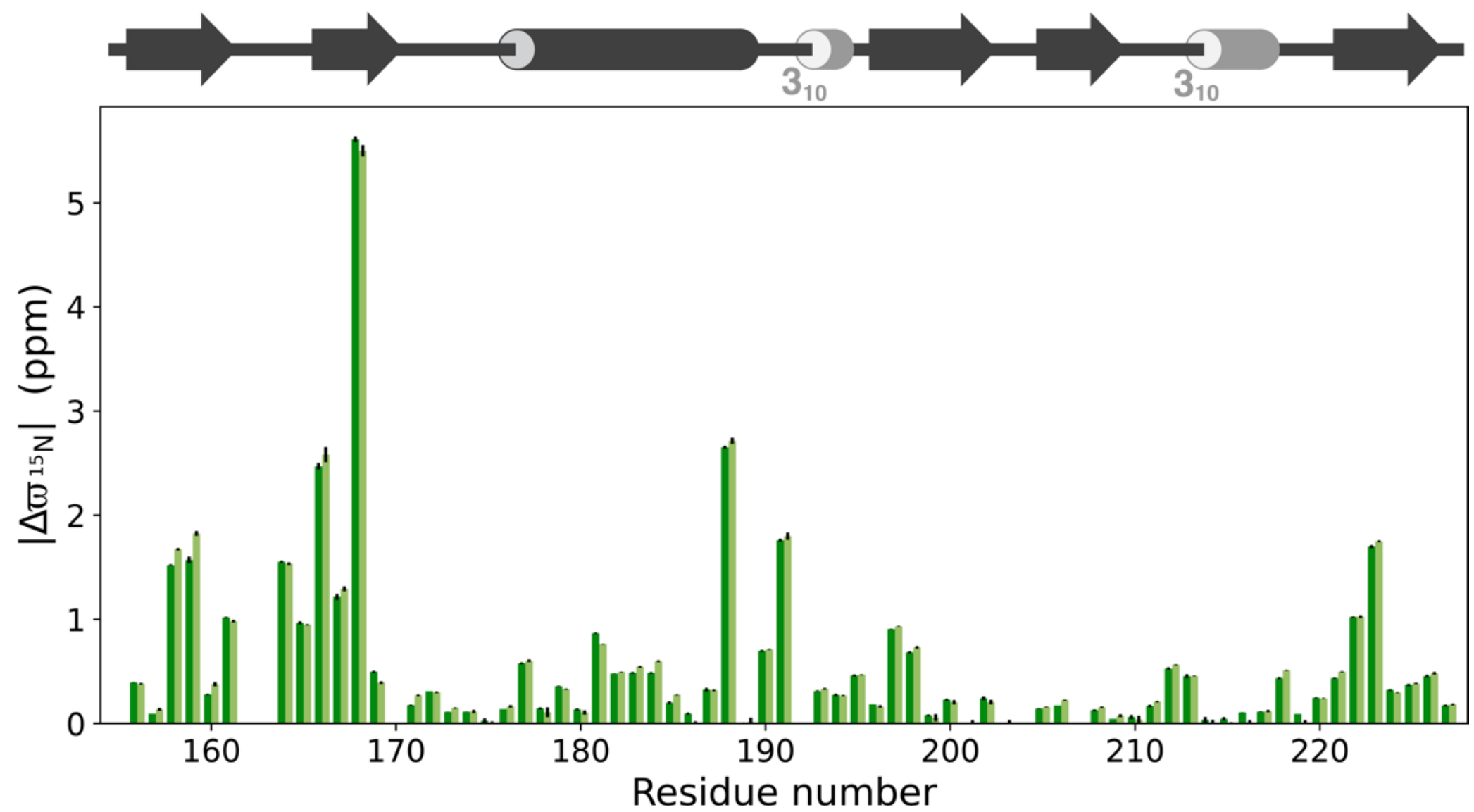

Figure S7. Absolute ^{15}N chemical shift differences $|\Delta\varpi_N|$ between RBD in the K-Ras•GDP-bound state and free state derived from ^{15}N-CEST and ^{15}N-CPMG experiments. $|\Delta\varpi_N|$ values of RBD interacting with WT K-Ras•GDP are in green and RBD interacting with G12D K-Ras•GDP in light green. The secondary structure of RBD is displayed on the top with helices represented by cylinders and β-sheets by arrows.

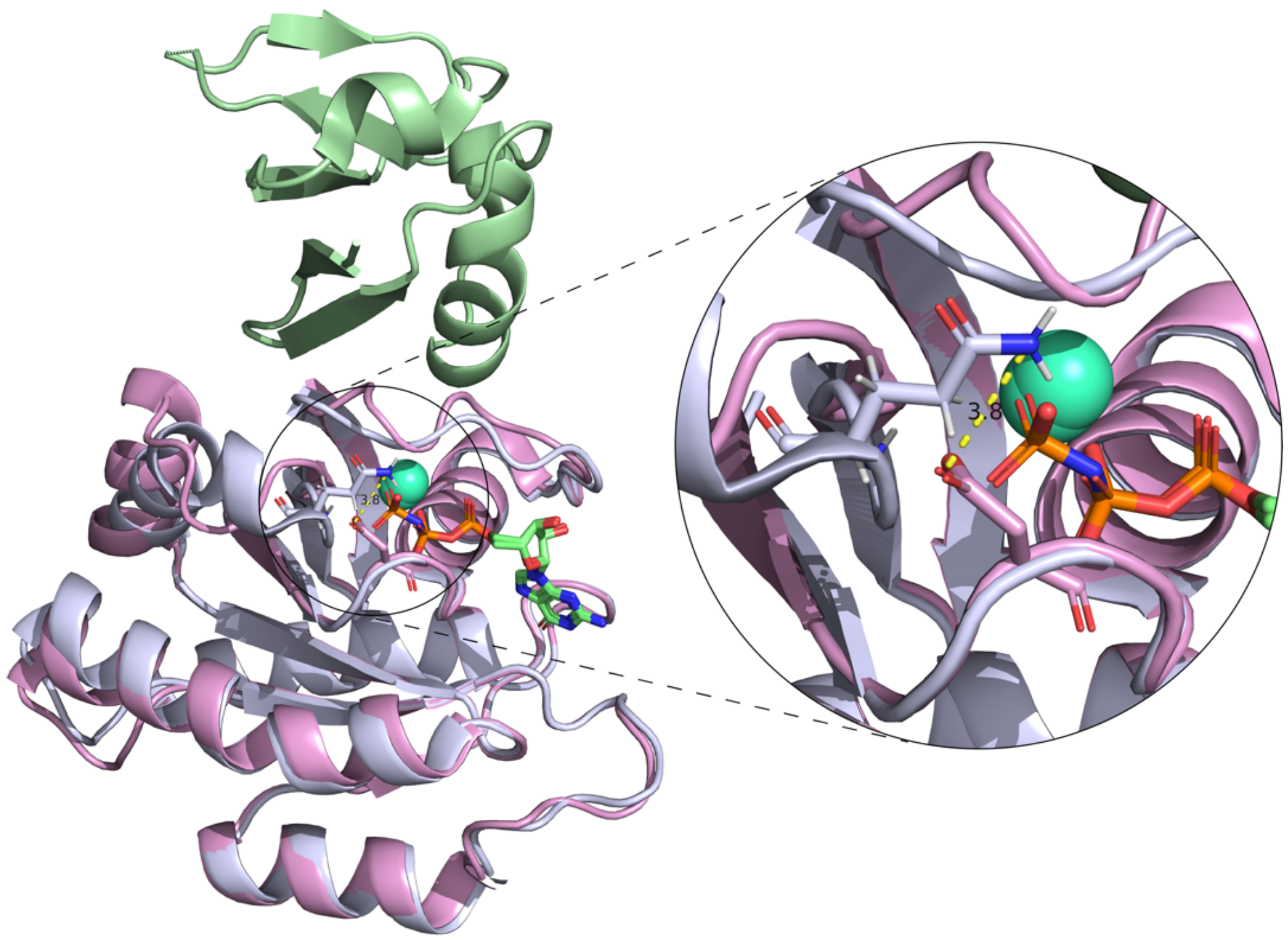

Figure S8. Cryo-EM and X-ray crystal structures of K-Ras•GNP•RBD complex and K-Ras•GDP. The cyro-EM structure of WT K-Ras•GNP•B-Raf-RBD complex is from PDB 8DGS, where K-Ras•GNP is colored in light blue and RBD in light green. The X-ray crystal structure of G12D K-Ras•GDP is from PDB 4EPR and is displayed in pink. Mg^{2+} ions are shown as teal spheres, and GDP and GNP nucleotides are represented as sticks. The G12D K-Ras•GDP structure is aligned with respect to the K-Ras•GNP part in the complex, and the side chains of residue Q61 of WT K-Ras•GNP and D12 of G12D K-Ras•GDP are highlighted as sticks and enlarged on the right. The side-chain NH_2 group of Q61 is in close proximity to the carboxylic acid in the side chain of D12, with a distance between amide nitrogen and carbonyl oxygen of 3.8 Å (yellow dashed line). This suggests that G12D K-Ras might form a hydrogen bond between the side chains of D12 and Q61 to help stabilize the conformation that more favorably binds to RBD.
